# Maximizing the potential of aggressive mouse tumor models in preclinical drug testing

**DOI:** 10.1038/s41598-021-91167-6

**Published:** 2021-06-02

**Authors:** M. Tarek Elghetany, Jia-Min Ho, Lois Hew Shi-Qi, Sekar Karthik, Jack M. F. Su, Qi Lin, YuChen Du, Jianhe Shen, Wing-Yuk Chow, Ching C. Lau, Adekunle Adesina, Angela Major, Anat Erdreich-Epstein, Kam-Man Hui, Xiao-Nan Li, Wan-Yee Teo

**Affiliations:** 1 Baylor College of Medicine, Houston, TX USA; 2 Department of Pathology, Texas Children’s Hospital, Houston, TX USA; 3 Humphrey Oei Institute of Cancer Research, National Cancer Center Singapore, Singapore, Singapore; 4 Pediatric Brain Tumor Research Office, SingHealth-Duke-NUS Academic Medical Center, Singapore, Singapore; 5 Division of Hematology-Oncology, Department of Pediatrics, Texas Children’s Cancer Center, Houston, TX USA; 6 Dan L. Duncan Cancer Center, Houston, TX USA; 7 Ann & Robert H. Lurie Children’s Hospital of Chicago, Chicago, IL USA; 8 Northwestern University Feinberg School of Medicine, Chicago, IL USA; 9 Connecticut Children’s Medical Center, The Jackson Laboratory for Genomic Medicine, University of Connecticut School of Medicine, Farmington, USA; 10 Department of Molecular Pathology, Texas Children’s Hospital, Houston, TX USA; 11 Departments of Pediatrics and Pathology, Children’s Hospital Los Angeles, Norris Comprehensive Cancer Center, and the Keck School of Medicine, University of Southern California, Los Angeles, CA USA; 12 Institute of Molecular and Cell Biology, A*STAR, Singapore, Singapore; 13 Cancer and Stem Cell Biology Program, Duke-NUS Medical School, Singapore, Singapore; 14 KK Women’s & Children’s Hospital, Singapore, Singapore

**Keywords:** Cancer, Drug discovery, Neuroscience, Diseases, Oncology

## Abstract

Atypical teratoid rhabdoid tumor (ATRT) is an aggressive embryonal brain tumor among infants and young children. Two challenges exist for preclinical testing in ATRT. First, genetically quiet, ATRT is a difficult tumor to target molecularly. Tumor cells need to divide to propagate tumor growth—intercepting the common crossroads in cell cycle progression is a feasible strategy. KIF11 is needed for bipolar spindle formation in metaphase. We identified KIF11 as a universal target of all ATRT-molecular-subtypes. Ispinesib, a KIF11-inhibitor, effectively inhibited tumor proliferation in all seven cell lines. A second challenge—a major challenge in preclinical drug testing in-vivo among aggressive tumor models, is the narrow therapeutic window to administer drugs within the limited murine lifespan. Our most aggressive ATRT tumor model was lethal in all mice within ~ 1 month of tumor implantation. Such short-surviving mouse models are difficult to employ for preclinical drug testing due to the narrow time window to administer drugs. To overcome this time restriction, we developed a clinical staging system which allowed physically-fit mice to continue treatment, in contrast to the conventional method of fixed drug-dose-duration regimen in preclinical testing which will not be feasible in such short-surviving mouse models. We validated this approach in a second embryonal brain tumor, medulloblastoma. This is a clinically relevant, cost-efficient approach in preclinical testing for cancer and non-cancer disease phenotypes. Widely used preclinical mouse models are not the most accurate and lack the aggressive tumor spectrum found within a single tumor type. Mice bearing the most aggressive tumor spectrum progress rapidly in the limited murine life-span, resulting in a narrow therapeutic window to administer drugs, and are thus difficult to employ in preclinical testing. Our approach overcomes this challenge. We discovered ispinesib is efficacious against two embryonal brain tumor types.

## Introduction

Widely used preclinical mouse models are not the most accurate and lack the aggressive tumor spectrum found within a single tumor type. Preclinical patient-derived orthotopic xenograft (PDOX) mouse models bearing the more aggressive tumor spectrum of a single tumor type, are difficult to employ for drug testing because of the short laboratory lifespan. Post-operative recovery after orthotopic brain tumor implantation requires 10–14 days^1^. For an aggressive mouse model which survives only 1 month post-tumor-implantation, it leaves a narrow time window to administer the drugs and the consequent challenge to achieve sufficient drug doses within the remaining 14–20 days of murine laboratory life-span to induce a tumor resolution. Therefore, the current way of conducting preclinical testing, uses less aggressive models within the tumor spectrum of a single tumor type, because the animals have a longer murine laboratory life span to allow a feasible time window to administer the drug. The main problem with this approach is that, in order to overcome this time window to administer the drug, researchers will employ less lethal models that are still representative of the tumor, but with a longer median survival. Consequently, aggressive mouse models bearing the greatest burden of aggressiveness on the tumor spectrum, are being underutilized in the field of preclinical testing.

Aggressive tumors in PDOX mouse models progress rapidly in the limited murine life-span^1,2^. Though these PDOX models are accurate representation and replicas of the original patients’ disease, they are not used because the mice succumb quickly in the disease course and this translates to a narrow therapeutic window to administer drugs in preclinical testing. Targeting the most aggressive models within the tumor spectrum is a high priority to efficiently curate the most effective drugs against a single tumor type. To enable us to use these highly aggressive mouse models, we developed a fitness-based staging method to closely monitor the mice health. This clinical staging system was necessary to allow physically-fit mice, while carrying the burden of an aggressive tumor spectrum, to continue receiving treatment. This fitness-based approach is in contrast to the conventional method of preclinical testing. In the conventional approach, all mice have to be administered the same number of drug doses over a designated duration. For mice xenografts carrying aggressive tumor spectrum types, they become sick rapidly and *not all* the mice in the cohort will be able to survive or tolerate all the drug doses in this conventional fixed drug-dose-duration regimen. Consequently, the healthier mice within the same cohort will have to stop treatment at the same time as their sicker counterparts, therefore losing the opportunity of receiving further drug treatment to evaluate the drug efficacy. This will render such aggressive mouse models not useful and hence not used for preclinical testing. In our current fitness-based approach, we allowed the healthier mice to continue on the treatment regimen, and the drug was able to prolong the survival of these animals who were fit to receive a longer treatment duration, reflecting a more accurate drug efficacy. This strategy allows aggressive mouse xenografts to be utilized for preclinical testing. This is clinically relevant because currently, these PDOX mice modelling an accurate aggressive tumor spectrum, are unfortunately not used for drug testing because of lifespan limitations. Omitting these aggressive models in preclinical testing further translates to not having drugs tested on the most aggressive spectrum of tumor type before moving drugs into clinical trials for patients.

We use an embryonal brain tumor in childhood, atypical teratoid rhabdoid tumor (ATRT) as an example to illustrate this challenge^3^. Pediatric brain cancer is the leading cause of death in childhood cancer^4,5^. ATRT is a rare tumor and most frequently affects infants and young children, accounting for 15–20% of all brain tumors in children less than 3 years of age^4,5^. It is an aggressive brain tumor and historical survival outcome is poor (10–20%, 5-year survival for patients < 3 years old)^6^. Standard of care involved surgery, chemotherapy and radiation therapy, although none are curative therapies^7^. A recently published trial ACNS0333 employing high dose chemotherapy and peripheral blood stem cell rescue^8^ has improved survival. However, this improved survival was achieved at the cost of treatment toxicity with high dose chemotherapy and the subsequent requirement for peripheral blood stem cell rescue^8^. High rates of treatment failure were observed in this trial^8^. More effective drugs are needed for ATRT. However, two challenges exist for preclinical testing in ATRT. First, lethal and genetically quiet^9^, ATRT is a difficult tumor to target molecularly. Tumor cells need to divide to propagate tumor growth—intercepting the common crossroads in cell cycle progression is a feasible strategy. KIF11 is needed for formation of bipolar spindle in metaphase^10^. In this study, we found KIF11 enriched among ATRT tumors, and ispinesib (a KIF11 inhibitor) was an efficacious agent for this tumor type. A second challenge—a major challenge in preclinical drug testing in-vivo among aggressive tumor models, is the narrow therapeutic window within limited murine lifespan. We developed a large panel of PDOX ATRT models—the most aggressive PDOX ATRT model has a laboratory murine life-span of about 1 month. Post-operative recovery after orthotopic brain tumor implantation requires 10–14 days^1^, which leaves a narrow time window to administer the drugs and the consequent challenge to achieve sufficient drug doses within the remaining 14–20 days of murine laboratory life-span of this aggressive PDOX model to induce a tumor resolution. Which in this case, renders majority of these aggressive preclinical models not useful to be employed in the laboratory. Our fitness-based staging method addressed this problem. Our goal is to allow mouse models bearing the most aggressive tumor spectrum to undergo preclinical testing in order to identify effective drugs against the most aggressive tumor on the spectrum, to translate to patient care and ultimately impact the survival of these patients who are failing current standard therapy.

The problem we address here, is the inability to use these valuable models of the most aggressive spectrum for preclinical testing due to the fitness limitations. Our fitness-based staging system is derived to overcome this limitation. Mice which are healthier, will be able to tolerate additional drug doses during our preclinical testing phase, and by staging their fitness level, we are able to identify and deliver more drug doses to these mice to evaluate the true drug efficacy, instead of a shortened drug treatment due to their reduced laboratory lifespan. This method enabled us to circumvent the laboratory problem of employing such preclinical models of aggressive tumors, with very short laboratory life span, hence a narrow drug delivery time window. We applied and validated *the method* of fitness-based staging in preclinical testing, *the model/s* of most aggressive tumor spectrum of embryonal brain tumors, and *the drug* ispinesib in this study.

## Results

### Targeting the most aggressive PDOX mouse model of ATRT

**Figure 1:**
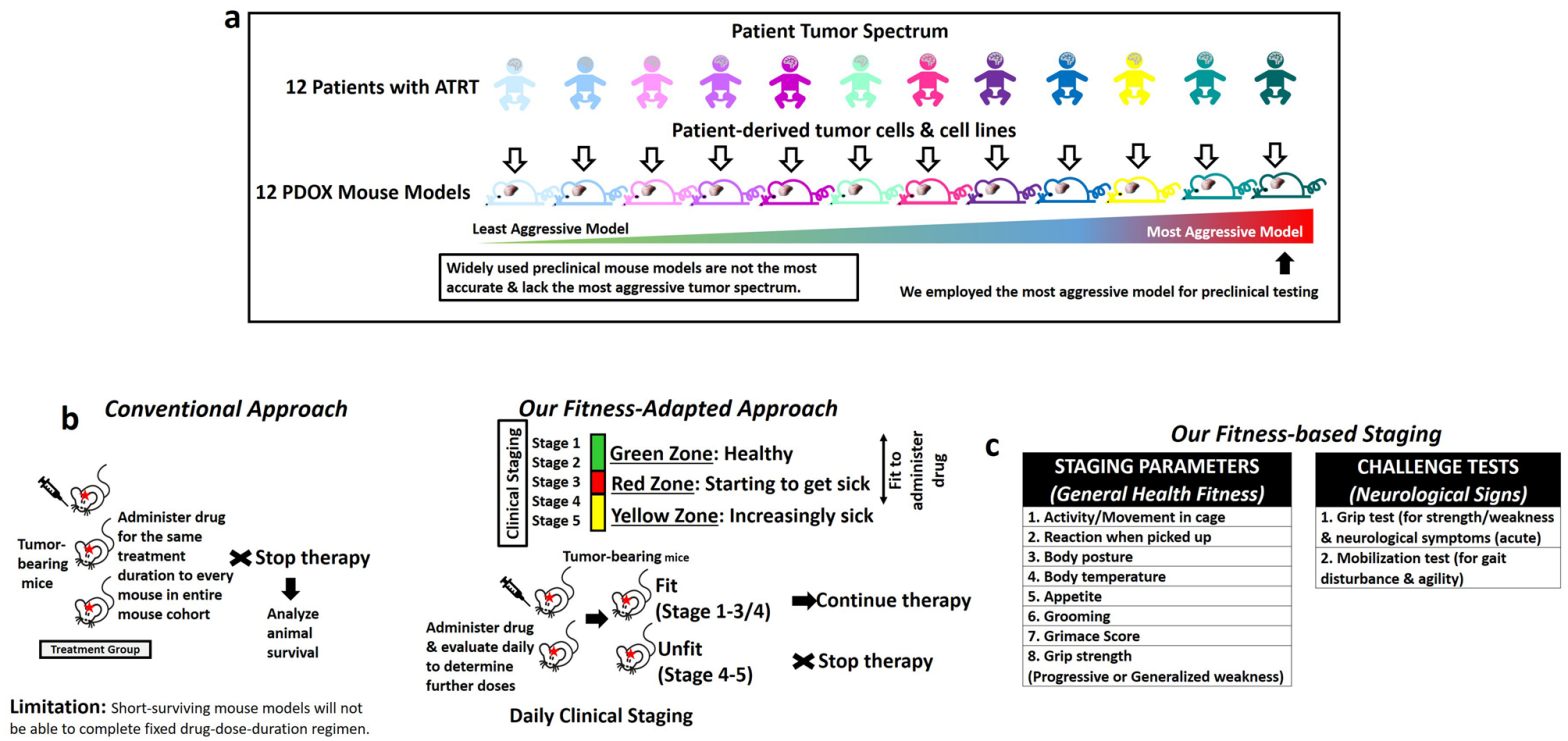
Fitness-based preclinical testing approach employing tumor model of the most aggressive tumor spectrum. (**a**) Widely used preclinical mouse models are not the most accurate and lack the aggressive tumor spectrum found within a single tumor type. Our fitness-based approach enabled us to employ the most aggressive tumor model for preclinical testing despite the short murine laboratory life-span. (**b**) Schematic illustrating *conventional approach* of administering drug for the same duration in each mice of the treatment cohort, in comparison to our current *fitness-adapted approach* of clinically staging the mice, matching their suitability to continue on ispinesib treatment regimen. (**c**) Eight staging parameters for general health fitness, and challenge tests to elicit neurological signs.

We have developed a large panel of PDOX mouse models using patient tumors and patient-tumor-derived cell lines of ATRT (Figs. 1a, S1A,B). The most aggressive ATRT tumor model we have developed was invariably lethal in all mice within ~ 1 month of tumor implantation, across multiple reproducible cohorts of mouse xenografts (Figs. 1a, S1B). Drug delivery to this tumor model was restricted by the rapid tumor progression time-line.

### Derivation of a clinical staging system for mice health fitness

**Figure 2:**
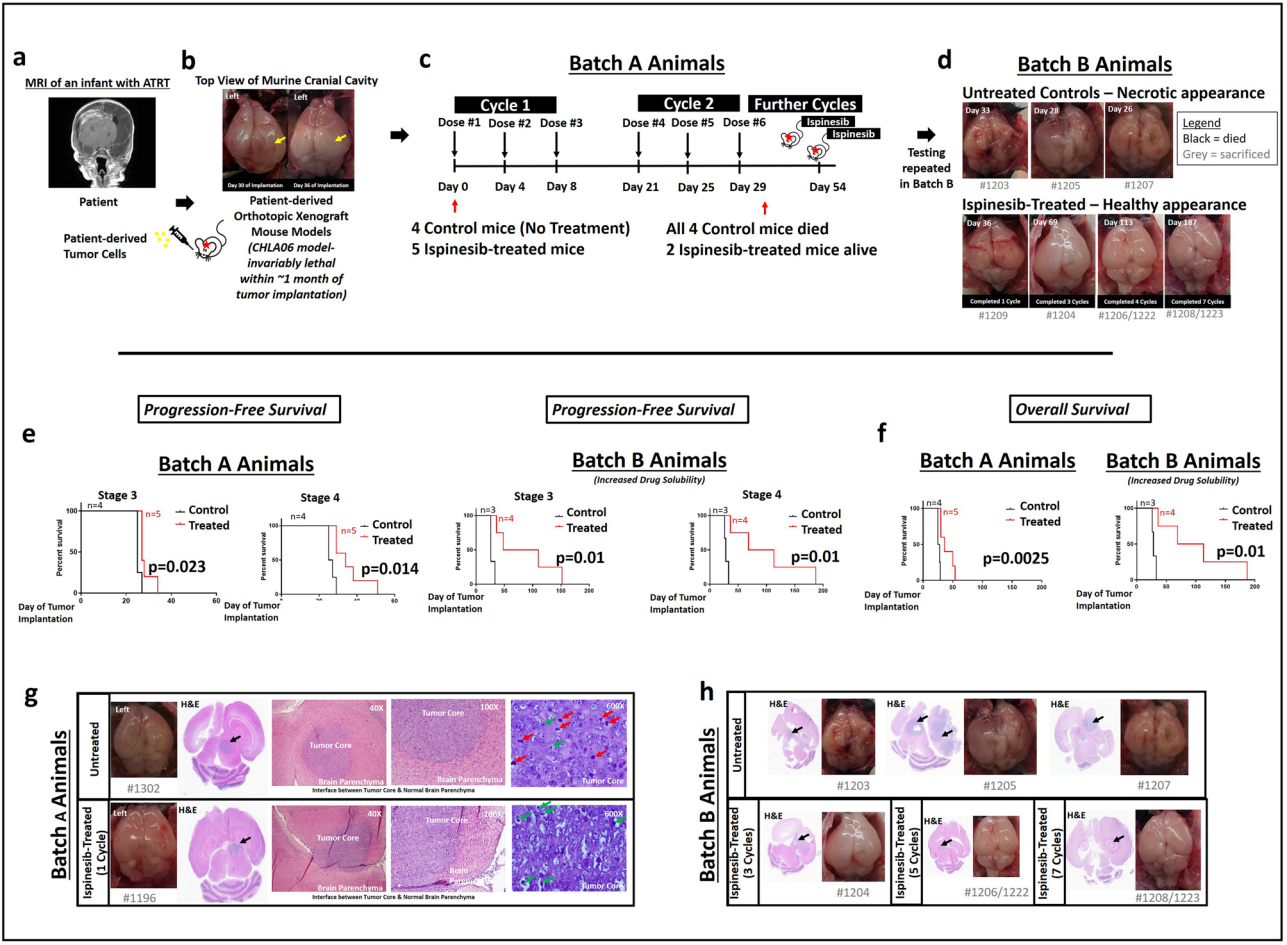
Ispinesib is a promising therapy for atypical teratoid rhabdoid tumor. (**a**) Coronal section of magnetic resonance imaging (MRI) of an infant with atypical teratoid rhabdoid tumor (ATRT). (**b**) Top view of cranial cavity of different mice implanted with CHLA-06 tumor cells into the right cerebrum (*yellow arrows* highlight the bulging right brain with tumor growth beneath). (**c**) Treatment timeline in Batch A animals (Treatment Group = 5, Control Group = 4). Ispinesib in-vivo dosing was 10 mg/kg administered intraperitoneally every 4 days for three doses, with the treatment course repeated on day 21. (**d**) Top view of cranial cavity of mice brains in Batch B animals (Treatment Group = 4, Control Group = 3). Brains from ispinesib-treated animals (*lower panel*) were structurally more normal in appearance, in contrast to untreated control animals (*upper panel*) which were distorted by tumor growth and more necrotic in appearance. Similar findings were observed in Batch A animals (Fig. S5). (**e**) Progression-free survival of animals in both Batch A and Batch B demonstrating significantly improved survival outcome among ispinesib-treated mice (Log-rank test, Mantel-Cox method). Solubility of ispinesib was increased using different diluent for reconstitution (“Materials and methods”, Batch A: Less soluble. Batch B: More soluble). (**f**) Overall survival of animals in both Batch A and Batch B similarly demonstrating significantly improved survival outcome among ispinesib-treated mice (Log-rank test, Mantel-Cox method). (**g**) Hematoxylin and eosin (H&E) staining of formalin-fixed mouse brains (ispinesib-treated versus untreated control, Batch A animals). Coronal sections of mouse brains from ispinesib-treated versus untreated mice showing a tumor mass (*black arrows*) in the center compressing against normal brain tissue. Magnification (×40 and ×100) photomicrographs showing tumor core interfacing with normal brain parenchyma in mice brains. Magnification (×600) photomicrographs showing ispinesib-treated tumors demonstrating frequent apoptotic bodies (*green arrows*) but no mitoses (*red arrows*). In contrast, untreated control xenograft tumors demonstrated numerous mitoses (*red arrows*) and a small number of apoptotic bodies (*green arrows*). (**h**) H&E Coronal sections of ispinesib-treated versus untreated control (Batch B animals). *Black arrows* indicate tumor. Absence of a visible tumor in mouse which received 7-cycles of ispinesib. Effects were tumor-specific; surrounding mouse brain parenchyma remained healthy with normal appearance, after 7 cycles of ispinesib.

**Figure 3:**
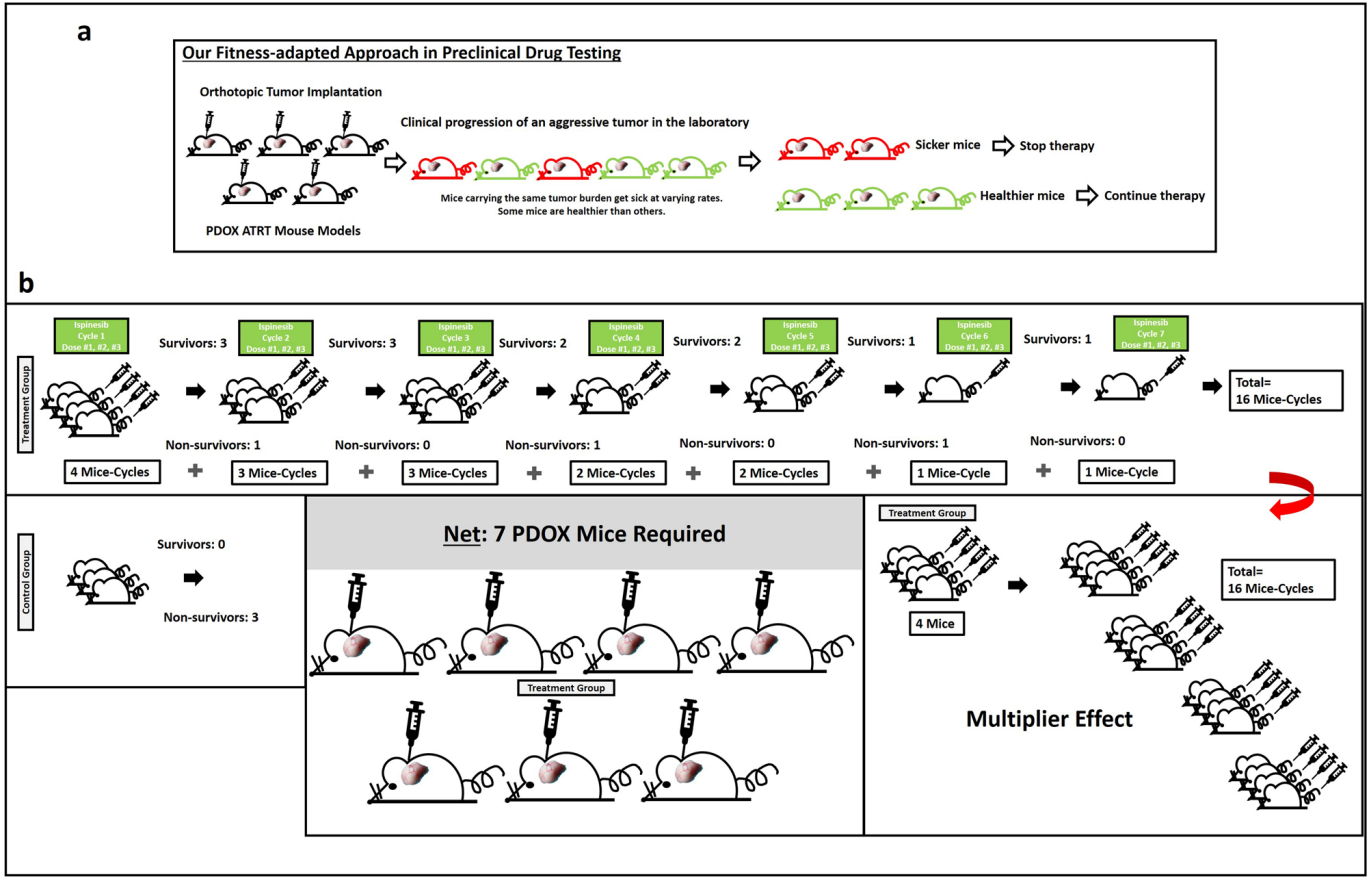
Parallel approach to clinic setting. (**a**) Our fitness-based staging method leverages the concept that mice health status varies despite carrying the same tumor burden. Mice which are healthier, will be able to tolerate additional drug doses during our preclinical testing phase, and by staging their fitness level, we are able to identify and deliver more drug doses to these mice to evaluate the true drug efficacy, instead of a short drug treatment due to their reduced laboratory lifespan. This method enabled us to circumvent a laboratory problem employing such preclinical models of aggressive tumors, with very short laboratory life span, hence a narrow drug delivery time window. Aggressive brain tumors have rapid clinical progression in patients. Some patients become sick more quickly while others remain in overall good health over the treatment course (*for illustration purpose only, ratio of healthier versus sicker patients varies in different settings)*. Patients in good overall health status will be fit to receive further cycles of therapy. In contrast, sicker patients will not be able to tolerate further therapy. (**b**) Multiplier effect of our fitness-based approach necessitated only 4 PDOX mice in the treatment group (example shown here for Batch B experiment), yielding a net 16 mice-cycles of drug treatment. This lessened animal numbers for drug testing, hence reducing the cost of preclinical studies. Further, our approach enabled healthier mice to receive a higher cumulative drug dose over a longer time period, to evaluate the in-vivo drug efficacy against the tumor. A statistically significant improvement in progression-free and overall survival was achieved using small animal cohorts, and this effect was replicated (Batch A and Batch B).

The conventional method of administering a drug for the same fixed drug-dose-duration regimen to every mouse in the entire mouse cohort (Fig. 1b,c), will not be a feasible approach for preclinical testing in this aggressive PDOX mouse model with a very short laboratory murine life-span of ~ 1 month due to its rapid health deterioration (Fig. 2a,b). Post-operative recovery after orthotopic brain tumor implantation requires 10–14 days^1^, which leaves a narrow time window to administer the drug and the consequent challenge to achieve sufficient drug doses within the remaining 14–20 days of murine laboratory life-span of this aggressive PDOX model to induce a tumor resolution. Ispinesib treatment has to be administered one dose every 4 days for three doses, with the treatment course repeated on day 21 (Figs. 2c, 3a). This will equate to only 3 doses of ispinesib within the remaining 14–20 days of the murine laboratory life-span after post-operative recovery using the conventional approach of fixed drug-dose-duration regimen, because the animals will invariably die from the aggressive tumor in 1 month without any treatment. It will be possibly unlikely that these 3 doses of an efficacious drug will be able to execute a rapid tumor resolution in that short span of time. To enable us to use this aggressive model, we derived a clinical staging system to closely monitor the health fitness of mice (Fig. 1b,c), concurrently matching their suitability to continue on ispinesib treatment regimen, Fig. 1a illustrates the necessity of the fitness score while simultaneously using a model representative of the most aggressive state of tumorigenesis.

Paralleling their human disease counterparts, some mice were physically fitter than others during the treatment course (Figs. 1b, 2c, 3a, S2–4), despite uniformly carrying the same tumor burden (same cell dose) from orthotopically implanted CHLA-06 ATRT cells (Fig. 2a,b). CHLA-06 was the most aggressive phenotype in our panel of ATRT tumor models (Figs. 2a,b, S1A,B). The growth patterns of the same tumor in each mouse can differ and result in different neurological deficits, compromising the health status of the mice to different extent. Hence some mice can be healthier and others are sicker. This observed fitness spectrum among the mice cohort mirrored the varied health fitness status in the clinical care of each individual patient with the same tumor type (Fig. 3a). Health fitness and neurological deficits of each patient can vary despite carrying the same brain tumor. We staged the mice from Stage 1 to 5 on a daily basis (5 days/week), using clinical categories of 8 parameters (Figs. 1c, S3A) including grimace score^11^, activity, reaction to handling, body posture, appetite, grooming and grip strength. Additional challenge tests (Figs. 1c, S3B) such as grip test (for strength and neurological symptoms) and mobilization test (for gait disturbance, agility) will elicit neurological signs.

Our clinical staging system was able to monitor progressive weakness and differentiate it from acute neurological deficits. In creating PDOX brain tumor mouse models^1,2^, we found these parameters most useful in determining the general health and neurological deficits of mice. Mild neurological signs related to the effects of brain tumor, impairing agility, focal weakness and gait disturbance (not affecting animal overall health fitness and function) were classified under a separate category of neurological deficits (Figs. 1c, S3A,B). These mild deficits were not included in the staging criteria. Progressive or generalized weakness affecting general well-being of the animals (using the 8 staging parameters) was included in the staging criteria. We targeted this window of health fitness in the mice and continued administering ispinesib to the physically-fit animals (between Stage 1 to 3/4, Figs. 1b, 2c).

### Ispinesib treatment using a fitness-based approach improved animal survival

Strikingly, using this clinical staging system to evaluate animal health fitness and determine their suitability to continue drug therapy, many of these animals survived to continue receiving additional cycles of ispinesib (Figs. 2c–f, S2). This approach significantly prolonged the overall survival of ispinesib-treated mice compared to untreated controls (Fig. 2f, Batch A animals, p = 0.0025, Log-rank test), despite small animal numbers. Ispinesib tripled the median survival in a repeated batch of animals treated using this fitness-based clinical staging system (Fig. 2f, Batch B animals, p = 0.010, Log-rank test). Drug solubility of ispinesib was increased using a different diluent for drug reconstitution in Batch B animals (see “Materials and methods”).

Additionally, this clinical staging approach was similarly useful in capturing the progression-free survival of mice (survival time from tumor implantation to Stage 3 and 4, Figs. 2e, S4) and demonstrated an improved progression-free survival with ispinesib therapy. Among Batch A animals which received 2 cycles of ispinesib therapy, one mouse brain did not display obvious tumor burden on serial sectioning with subsequent hematoxylin and eosin staining (Fig. S5B). Our fitness-adapted approach of drug administration yielded a multiplier effect by enabling more mice to undergo additional cycles of therapy (a treatment group of 4 mice with a net effect of 16 mice-treatment-cycles over 7 drug cycles, Figs. 3b, S2B) in contrast to the conventional approach of fixed drug-dose-duration regimen, whereby the drug is administered over the same fixed duration for every mouse in the treatment cohort (Fig. 1b).

### KIF11 is a universal target of all 3 ATRT subtypes

**Figure 4:**
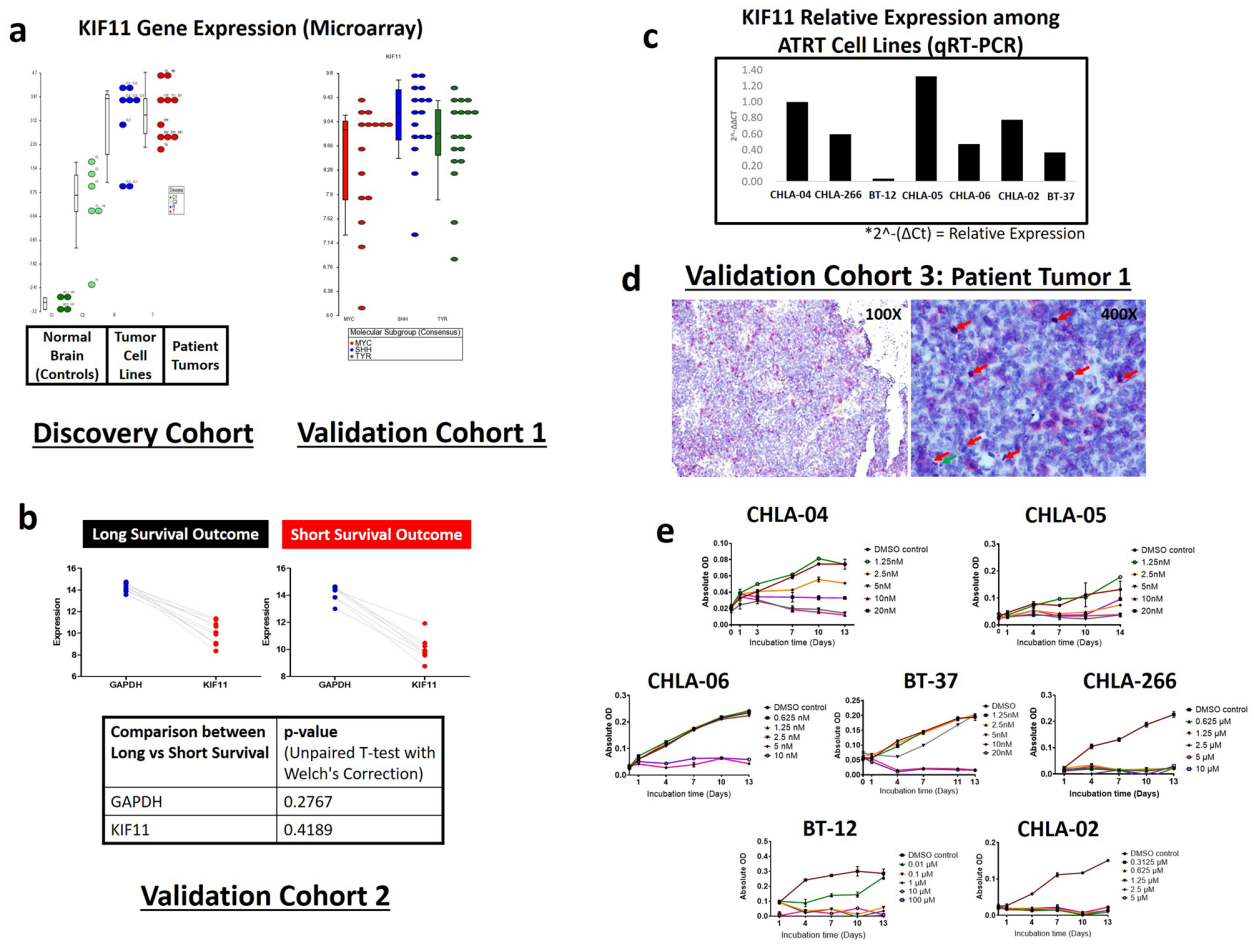
KIF11 is a universal target across all ATRT-subtypes and KIF11-targeting with ispinesib inhibited proliferation in a panel of 7 ATRT cell lines. KIF11 gene expression in (**a**) Discovery Cohort of 10 patient tumors, 8 patient-derived tumor cell lines, 10 normal brain control tissues (RNA-Seq), Validation Cohort 1 of patient tumors of three ATRT subtypes—SHH-ATRTs, MYC-ATRTs and TYR-ATRTs (n = 49, Microarray Affymetrix U133plus2.0, GSE70678^18^), (**b**) Validation Cohort 2 of 18 patient tumors with long or short survival outcome (GSE28026^19^, Affymetrix U133plus2.0), and (**c**) KIF11 relative gene expression in our panel of 7 patient-derived ATRT cell lines. For Discovery Cohort, KIF11 was highly expressed in patient tumors (Fold change 24.4, p < 0.0001, Student T-Test) and tumor cell lines compared to normal brain controls. For Validation Cohort 1, patient tumors of all 3 molecular ATRT-subtypes exhibited high KIF11 expression, with SHH-ATRTs demonstrating the highest KIF11 expression compared to MYC-ATRTs and TYR-ATRTs (fold change 1.4, p = 0.04, Student T-Test). For Validation Cohort 2, KIF11 expression was referenced to GAPDH (housekeeping gene), KIF11 was highly expressed in patient groups of both long and short survival outcome (p = 0.4, unpaired T-test with Welch's Correction). (**d**) Immunohistochemistry of patient tumors from Validation Cohort 3 similarly demonstrated KIF11 expression, showing Patient Tumor 1 with approximately 10–20% staining (*red arrows* indicate mitoses, *green arrows* indicate apoptotic bodies). KIF11 protein expression co-localized with mitoses. KIF11 (target of ispinesib) was expressed on different cell populations within patient tumor, and not restricted to mitoses, indicating ispinesib targeted not only mitoses (actively dividing cells) but also other tumor cell populations within the tumor. (**e**) Targeted KIF11 inhibition: Dose–response effect of ispinesib on 7 ATRT cell lines. Values are shown as gradations of green (lowest dose) to purple (highest dose).

Genetically quiet^9^, ATRT is a difficult tumor to target molecularly. SMARCB1 mutation is the only consistent mutation that has been more frequently observed^12,13^. KIF11 is needed for formation of the bipolar spindle in metaphase^10^. KIF11 suppression leads to prolonged mitotic arrest and subsequent cell death in mitosis^10^. KIF11 inhibitors are in Phase II trials for ovarian cancer^14^, and other adult advanced solid tumors^14^ such as breast, colorectal, renal, and lung cancers, head and neck squamous cell carcinoma, hepatocellular carcinoma and melanoma^15^, and Phase I trial for pediatric brain and solid tumors^16,17^. Ispinesib, a KIF11 inhibitor, has demonstrated effective blood brain barrier penetration in glioblastoma xenografts^10^. We found KIF11 overexpressed in a Discovery Cohort of 10 patient tumors, 8 tumor cell lines of ATRT, compared to 10 normal brain tumor tissues (Fig. 4a). Importantly, in Validation Cohort 1, KIF11 was highly upregulated in a total of 49 patient tumors of all three molecular subtypes of ATRT^18^ (MYC-ATRTs, TYR-ATRTs, and SHH-ATRTs, Fig. 4a, GSE70678^18^, Fig. S6A). This makes KIF11 a universal target of all ATRT-subtypes. KIF11 was similarly highly expressed in Validation Cohort 2 of 18 patient ATRT tumors (Fig. 4b, GSE28026^19^). KIF11 expression among tumors from patient groups of long compared to short survival outcome (Validation Cohort 2, Fig. 4b) was similarly high (no statistical difference in expression level, p = 0.41, unpaired T-test with Welch's Correction), which was in consonance with our finding of universal high KIF11 expression of all 3 molecular ATRT-subtypes (Fig. 4a). In Validation Cohort 3, KIF11 protein was highly expressed on patient tumors on immunohistochemistry (Figs. 4d, S6B). KIF11 mRNA and protein were also highly expressed in ATRT cell lines (Figs. 4c, S6C).

### In-vitro ATRT cell proliferation inhibited by ispinesib

There are > 20 high affinity, specific small-molecule KIF11 inhibitors, including ispinesib^10^. We found ispinesib an efficacious drug against all 7 ATRT cell lines tested (Figs. 4e, S7C one additional ATRT cell line in our collection was very slow-growing and not suitable for proliferation assays-ATRT95). These 7 ATRT cell lines were cultured in their respective standard growth media which included serum-enriched as well as growth factors-enriched cancer stem cell media (Figs. 4e, S7C). Ispinesib was effective against tumor cells in the different growth conditions. This may additionally indicate the efficacy of ispinesib against cancer stem cell population of ATRT cells growing as neurospheres in-vitro in growth factors-enriched cancer stem cell media (Figs. 4e, S7C). Tumor cell proliferation was effectively inhibited between IC_50_ range of 4.5 nM–527 nM on Day 7 for all 7 cell lines (Fig. S7C). Ispinesib concentration of 5 nM–1.25 µM demonstrated sustained time-dependent killing-effect on all 7 cell lines over 13 days (Fig. S7C).

### ATRT cells undergo ispinesib-induced cell cycle arrest and apoptosis

**Figure 5:**
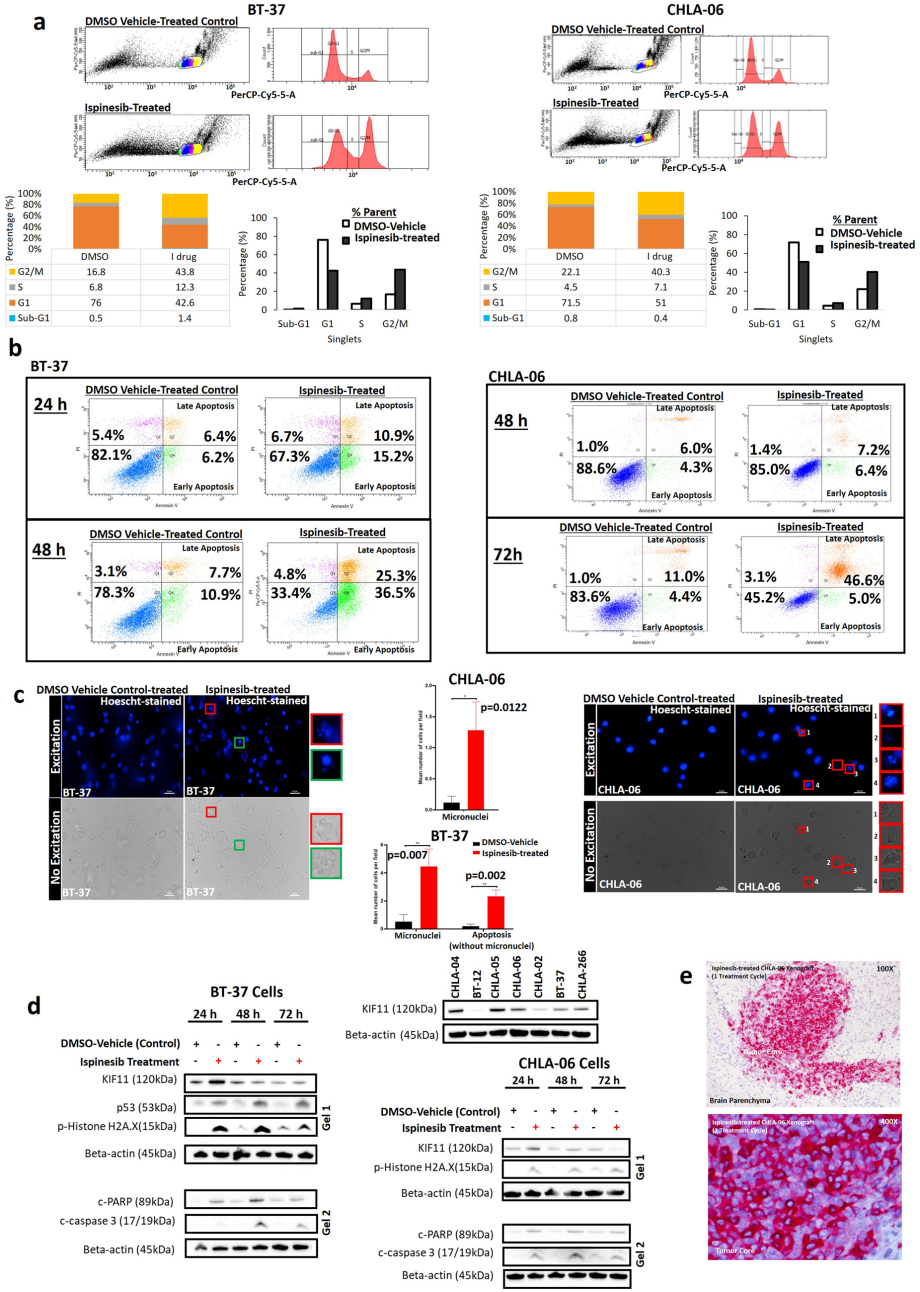
Functionally defined cell death mechanisms in ispinesib therapy. (**a**) Cell cycle analysis of CHLA-06 and BT-37 treated with ispinesib 4.69 nM (Day 4 IC_50_ dose) and 10 nM (Day 4 IC_90_ dose) respectively for 24 h. (**b**) Flow cytometry analysis for apoptotic cell death (Annexin V^+^ PI^−^) in BT-37 treated with ispinesib (10 nM) for 24 h and 48 h. Flow cytometry analysis for apoptotic cell death (Annexin V^+^ PI^−^) in CHLA-06 treated with ispinesib (4.6 nM) for 48 h and 72 h. (**c**) Hoechst staining of cell nuclei, BT-37 and CHLA-06 treated with 10 nM and 4.69 nM ispinesib respectively for 24 h. Ispinesib-treated BT-37 cells (average apoptotic cells = 2/hpf, average micronuclei = 4/hpf) demonstrated tenfold increase in apoptotic cells (p = 0.002, unpaired T-Test) and eightfold increase in micronuclei (p = 0.007, unpaired T-Test) compared to DMSO vehicle control treated cells (average apoptotic cells = 0.2/hpf, average micronuclei = 0.5/hpf). Ispinesib-treated CHLA-06 cells (average micronuclei = 1.28/hpf) demonstrated tenfold increase in micronuclei (p = 0.0122, unpaired T-Test) compared to DMSO vehicle control treated cells (average micronuclei = 0.117/hpf). Micronuclei (*red circle*) and condensed apoptotic nuclei (*green circle*) can be seen after ispinesib treatment. (**d**) p-Histone H2A.X was increased with 24 h and 48 h-ispinesib treatment in CHLA-06 and BT-37 cells, indicative of ispinesib inducing DNA-damage. c-caspase 3 and c-PARP were also increased with ispinesib treatment, supporting activation of intrinsic apoptosis pathway. (**e**) Immunohistochemistry demonstrates that in-vivo, tumor cells were enriched in KIF11 protein expression within tumor core of CHLA-06 xenograft (after 1 cycle of ispinesib treatment) in contrast to normal brain parenchyma which was bland for KIF11.

Ispinesib at ≤ 10 nM is highly effective against 2 most tumorigenic cell lines (CHLA-06, BT-37), inducing G2/M arrest (Fig. 5a) and causing apoptotic cell death (61.8% early and late apoptotic cells in BT-37 cells at 48 h, 51.6% in CHLA-06 cells at 72 h of ispinesib treatment, Fig. 5b). This apoptotic cell death effect was consistently observed with ispinesib treatment when tested on additional ATRT cell lines (Fig. S7). On hoechst-staining under fluorescent microscopy, ispinesib-treated BT-37 and CHLA-06 cells at 24 h (Figs. 5c, S6C, S8) demonstrated significant increase in micronuclei. Significant increase in apoptotic cells was also observed in ispinesib-treated BT-37 cells.

Immunoblotting (Figs. 5d, S6C,E) indicated diverse effects of ispinesib through a variety of cell death mechanisms on our collective panel of ATRT cell lines (CHLA-06, BT-37, CHLA-05). DNA damage was indicated by an increase in histone H2A.X in ispinesib-treated CHLA-06, CHLA-05 and BT-37 cells. KIF11 protein expression was enriched in CHLA-06, CHLA-05 and BT-37 cells after 24 h of ispinesib treatment. In-vivo, KIF11 protein enrichment was similarly observed after 1 cycle of ispinesib treatment (Fig. 5e). KIF11 can therefore, be a stable drug target. Ispinesib treatment upregulated caspase-3 and c-PARP protein expression in CHLA-06, CHLA-05 and BT-37 cells (Fig. 5d), supporting ispinesib-induced apoptosis.

We further confirmed the findings of ispinesib-induced apoptotic cell death in-vivo. Untreated CHLA-06 xenograft tumors (Fig. 2g) demonstrated numerous mitotic figures and few apoptotic cells, while ispinesib-treated xenograft tumors were almost devoid of mitotic figures and had frequent apoptotic cells. Effects were tumor-specific; surrounding mouse brain parenchyma remained healthy with normal appearance, after 7 cycles of ispinesib treatment (Fig. 2g,h). In patient tumors (Patient 1, Figs. 4d, S6B), we observed KIF11 expression co-localized with mitotic cells. Mitoses from our ATRT tumor model (Fig. 2g) were consistent with the representation of the typical distribution in the average patient tumor (Fig. 4d). KIF11 (target of ispinesib) was expressed on different cell populations within PDOX tumor and patient tumor (Fig. 4d), and not restricted to mitoses, indicating ispinesib targeted not only mitoses (actively dividing cells) but also other tumor cell populations within the tumor. KIF11 expression on actively dividing tumor cells, presented KIF11 as an attractive target for anti-cancer drugs. Taken together, ispinesib was an efficacious therapeutic agent against ATRT in-vitro (resulting in growth arrest and death of tumor cells) and in-vivo (leading to apoptotic tumor cell death and improved animal survival)*.*

### Ispinesib is efficacious in a 2nd pediatric embryonal brain tumor model

**Figure 6:**
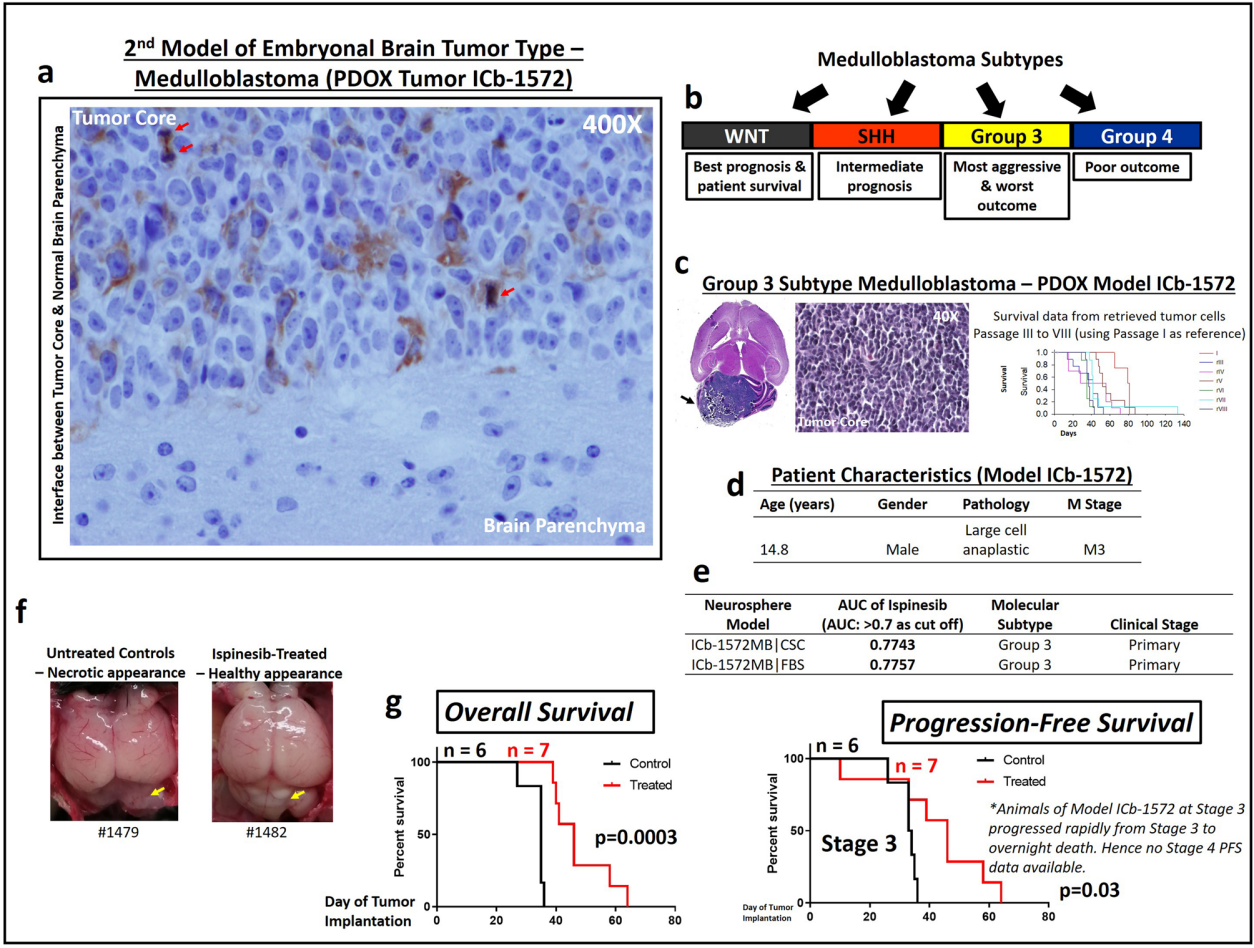
Ispinesib is efficacious against a second model of pediatric embryonal brain tumor. (**a**) KIF11 (target of ispinesib) was expressed on our PDOX models of medulloblastoma (MB), another type of embryonal brain tumor. MB is the most common malignant brain tumor in children. Immunohistochemical staining of several of our MB PDOX models with KIF11 antibody demonstrated KIF11 expression co-localized with mitotic figures, shown here in Model ICb-1572. KIF11 was expressed on different cell populations within PDOX tumor (shown here in *brown* staining), and not restricted to mitoses, indicating ispinesib targeted not only mitoses (actively dividing cells) but also other tumor cell populations within the tumor. Cytoplasmic staining of KIF11 was observed on 5–10% of cells, including mitotic figures (*red arrows*), ×400. Normal brain parenchyma was devoid of KIF11 expression, interfacing with tumor core densely packed with tumor cells. Actively dividing cells were marked by KIF11^+^ mitotic figures—a good target for ispinesib. (**b**) Pediatric MB is molecularly classified into 4 molecular subtypes: sonic-hedgehog (SHH)-activated MB, *Wingless* (WNT)-activated, and less characterized Group 3 and 4 subtypes^20,21^. Group 3 and 4 MBs are the most aggressive subtypes and Group 3 MBs have the worst survival outcome^22,23^. PDOX Model ICb-1572 is Group 3 subtype^24^. (**c**) Hematoxylin & eosin stains showing a large xenograft MB tumor of Group 3 subtype (Model ICb-1572), occupying almost the entire mouse cerebellum. Histological appearance (40X) of xenograft tumor was consistent with densely packed, small round blue cell typical of MB. Our panel of xenograft tumors recapitulate morphologic, histological and immunohistochemical features of matched patient original tumors^1^. Animal survival was monitored up to Passage IV-IX for Model ICb-1572. Log rank analysis of animal survival times of ICb-1572 MB during serial sub-transplantation of retrieved xenograft cells from cryopreservation (Passage III to VIII is shown here using Passage I as reference). Survival days were consistent and maintained within and across passages in mice. (**d**) Patient characteristics of Model ICb1572. (**e**) Tumor proliferation activity—ispinesib against in-vitro 3D-Neurosphere cultures of Model ICb1572. Cancer stem cell enriched neurospheres derived from aggressive Group 3 subtype (Model ICb-1572) demonstrated susceptibility to 7-day in-vitro treatment with ispinesib. Monolayer tumor cells of Model ICb-1572 demonstrated similar susceptibility. Area under curve (AUC) > 0.7 was the significant cut-off. (**f**) Top view of cranial cavity of mice brains (Treatment Group = 7, Control Group = 6). We employed the clinical staging system to evaluate animal health fitness and determine their suitability to continue ispinesib therapy. Brains, particularly the tumor-bearing cerebellum (*yellow arrows*), from ispinesib-treated animals (*right panel*) were structurally more normal in appearance, in contrast to untreated control animals (*left panel*) where the cerebellums were distorted by tumor growth, soft and necrotic in appearance. (**g**) Overall survival and progression survival (Stage 3) of animals was significantly improved with ispinesib treatment (p = 0.0003, p = 0.03 respectively, Log-rank test).

We observed KIF11 (target of ispinesib) expressed on some of our PDOX models of medulloblastoma (MB), another type of embryonal brain tumor^4,5^. MB is the most common malignant brain tumor in children^4,5^. In this second model of pediatric embryonal brain tumor, immunohistochemical staining of some MB PDOX models with KIF11 antibody demonstrated KIF11 expression co-localized with mitotic figures, illustrated by Model ICb-1572 (Fig. 6a). KIF11 was again expressed on different cell populations within PDOX tumor (Fig. 6a), and not restricted to mitoses, indicating ispinesib targeted not only mitoses (actively dividing cells) but also other tumor cell populations within the tumor. Cytoplasmic staining of KIF11 was observed on 5–10% of cells in Model ICb-1572, including mitotic figures (Fig. 6a, Magnification 400×). Normal brain parenchyma was devoid of KIF11 expression, interfacing with tumor core densely packed with tumor cells. Tumor core was enriched with actively dividing cells marked by KIF11^+^ mitotic figures (Fig. 6a), a good target for ispinesib. Pediatric MB is molecularly classified into 4 molecular subtypes: sonic-hedgehog (SHH)-activated MB, *Wingless* (WNT)-activated, and less characterized Group 3 and 4 subtypes (Fig. 6b)^20,21^. Group 3 and 4 MBs are most the aggressive subtypes with the worst survival outcome^22,23^. PDOX Model ICb-1572 is Group 3 subtype, characterized by genomic profiling (Fig. 6b–d)^24^. Our PDOX models have been characterized and recapitulated patient tumors^1,24^. Ispinesib effectively inhibited cancer stem cell enriched 3D-neurospheres derived from aggressive Group 3 PDOX MB (Model ICb-1572) over 7-day in-vitro treatment (Fig. 6e). Monolayer tumor cells of Model ICb-1572 demonstrated similar susceptibility (Fig. 6e). In-vivo, ispinesib significantly improved the overall survival (p = 0.0003, Log-rank test) of mice bearing Group 3 subtype MB (Model ICb-1572, Treatment Group = 7, Control Group = 6, Fig. 6f). We employed the same clinical staging system (Fig. 1b,c) to evaluate animal health fitness and determine their suitability to continue drug therapy. Brains, particularly the tumor-bearing cerebellum from ispinesib-treated animals (*right panel,* Fig. 6g) were structurally more normal in appearance, in contrast to untreated control animals (*left panel,* Fig. 6g) where the cerebellums were distorted by tumor growth, and were soft and necrotic in appearance.

## Discussion

Creation of PDOX models is a lengthy and costly process^25^. To derive sufficient statistical significance for survival analyses, large numbers of PDOX mice are required for preclinical testing of each drug. This further escalates the cost of in-vivo preclinical drug testing for each compound, using PDOX models. Nonetheless, PDOX models are still the preferred cancer model over culture-based spheroids or slice cultures^25^. In 2016, the US National Cancer Institute (NCI) has decided to stop screening most drugs using the NCI-60, its panel of 60 human cancer cell lines grown in culture, after more than 25 years of heavy use by worldwide researchers, and is developing PDOX models that better mimic the human counterpart. Aggressive models will curate the most efficacious drugs with strong preclinical data to translate into clinical trials for patients. However, these xenografts are under-utilized because a major limitation of such aggressive tumor models exists—the narrow time window for drug administration to gather statistically significant survival data in murine models with a limited laboratory life-span. Consequently, these aggressive tumor models are not the intuitive choice for preclinical drug testing.

Our current approach of staging the health fitness status in the mice, circumvents this problem and taps into the valuable resource of aggressive tumor models for therapeutic testing (Fig. S9, Schematic). Adopting this clinical staging system offered a unique opportunity to study drug efficacy in the most aggressive tumor models and unexpectedly decreased the cost of testing in PDOX models by using lesser animal numbers. Our fitness-adapted approach of drug administration yielded a multiplier effect by enabling more mice to undergo additional cycles of therapy in contrast to the conventional approach of fixed drug-dose-duration regimen. In the conventional approach, the drug is administered for the same fixed duration for every mouse in the treatment cohort which will not be feasible in aggressive PDOX mouse models with a very short laboratory murine life-span, with survival of ~ 1 month, due to their rapid health deterioration. Our strategy also enabled a higher total cumulative drug dose to be administered to some mice which were fit to receive more drug cycles, therefore maximizing the drug efficacy.

Preclinical studies and Phases I-III clinical trials are time consuming. A major factor contributing to clinical trial failure has been attributed to patient selection, as a therapeutic agent may only be effective in a subset of patients with the same cancer^25^. Targeting PDOX model of the most aggressive spectrum within each tumor type, which is our current approach, will strengthen the preclinical data and ensure that the drug brought to clinical trial will be efficacious against the most aggressive subset. As a collaborative group, our PDOX models have been characterized and recapitulate patient tumors^1,2,24^, and are widely used by the neurooncology community, and have led to subsequent clinical trials. Ispinesib is already in Phase II trials for many adult cancers^14,15^. Pharmacokinetic data for pediatric dosing of ispinesib is available from a prior small Phase I trial which included a mix of 24 pediatric recurrent/refractory solid tumors^16^. The available pharmacokinetic data will facilitate a pediatric Phase II trial for the two embryonal brain tumor types (ATRT and MB) evaluated for in-vivo efficacy in our study. Our preclinical data will be crucial for driving FDA approval of ispinesib for ATRT and MB.

Disadvantages of our approach include longer time required to perform daily clinical staging, more in-depth training and time invested in training research staff to perform accurate staging through observation, clinical examination and charting. Some may argue that using this fitness-based approach, different mice in the same treatment cohort will be administered different total drug doses, instead of a fixed total dose across all animals in the conventional approach. On the contrary, we would argue that our current approach provides more accurate data on the drug. During cancer treatment, patients differ in health fitness and their ability to tolerate drug treatment. Our approach recapitulates the varied clinical setting among patients with aggressive tumors, who will unavoidably receive adjustment in doses and treatment duration, particularly during clinical trial phase. Lastly, immunosuppressed mice used for xenograft modelling are highly vulnerable to infections and succumb quickly from common side effects of drugs, such as gastrointestinal effects and diarrhea. This can further increase animal number requirements and cost of preclinical testing in mice. Our approach of clinically staging the health status of mice will allow us to capture the variation in their health fitness and avoid drug administration in animals with poorer fitness, allowing recovery before proceeding with further drug doses. This will ensure that the animal survival will more accurately reflect the drug-on-tumor effects, instead of confounders such as drug side effects on immunocompromised mice leading to infections and thus shortening the animal life-span. Consequently, these animals can recover and continue on to receive a longer course of treatment to evaluate the highest drug efficacy.

PDOX models that accurately recapitulate the biology of corresponding patient tumors, will replicate the clinical course and survival patterns of the patient tumors^1,2,24,25^. Genomic profiling has subclassified each brain tumor type into different molecular subtypes, for example MB has been classified into 4 major molecular subtypes—SHH-subtype, WNT-subtype, Group 3 and Group 4 subtypes^20,21^. Group 3 and Group 4 are the most aggressive subtypes with the worst prognosis and survival outcome among patients^22,23^. Capability to use aggressive PDOX models for preclinical testing should be valued as an added advantage and an additional tool for preclinical testing. Our fitness-based approach of preclinical testing provides this added capability. Our fitness-based staging approach is feasible in both less aggressive models with longer laboratory life-span (data not shown), and more aggressive models with shorter laboratory life-span. We performed this fitness-based staging for up to 400–500 brain-tumor-bearing mice over the past few years. This provides further validation that *the method*, *the model/s* and *the drug*, are robust.

Majority of patients with ATRT are young children or infants < 3 years of age^6^. The standard of care, historically for this young age group is maximal tumor resection where safely possible^7^ and chemotherapy. Radiation therapy is often avoided in this young age group to avoid long term neurocognitive sequelae^7^. Outcome is dismal^6^ for this age group with the restriction of radiation therapy use. A recently published clinical trial ACNS0333 employing high dose chemotherapy and peripheral blood stem cell rescue^8^ has remarkably improved the 4-year event-free survival to 37% compared to historical cohort of 6.4%. However, this improved survival was achieved at the cost of treatment toxicity of high dose chemotherapy and the subsequent requirement for peripheral blood stem cell rescue^8^. Further, 91% of patients experienced treatment failures, which occurred by 2 years from enrollment^8^. More effective drugs are needed for ATRT. Ispinesib thus offers a potential promising therapeutic adjuvant post-surgery, particularly in the setting of minimal residual tumor, for tumor control and to improve patient survival. We tested ispinesib on 7 ATRT cell lines, with different growth rates—Ispinesib was effective in all 7 ATRT cell lines with different growth rates. This serves as additional support that ispinesib was a robust drug, efficacious among ATRT cells with different growth rates. Ispinesib was similarly efficacious in our second PDOX model of pediatric embryonal brain tumor—aggressive Group 3 MB. The gene target of ispinesib, KIF11, co-localized with mitotic figures in both ATRT and MB, which makes this drug a good target against actively dividing tumor cells. Additionally, KIF11 was expressed on different cell populations within PDOX tumor and patient tumor of both types of embryonal brain tumors (ATRT and MB), and not restricted to mitoses, indicating ispinesib targeted not only mitoses (actively dividing cells) but also other tumor cell populations within the tumor.

Finally, our approach in preclinical testing offers a threefold benefit: first, cost reduction in preclinical testing. Second, we tap into the valuable resource of aggressive tumor models for therapeutic testing, to curate the most efficacious drugs with robust preclinical strength to translate into clinical trials. Third, because aggressive tumors progress quickly such as our current CHLA-06 tumor model and ICb-1572 Group 3 MB model, the mice invariably die in a month—it provides in-vivo data in a short time-frame as any survival benefit prolonging murine lifespan will be rapidly evident in the laboratory.

## Materials and methods

### Ethics approval

All primary tumor specimens were obtained through an Institutional Review Board approved protocol from patients treated at the Texas Children’s Hospital (Houston, Texas, USA) after informed consent was signed. Paraffin sections from resected ATRTs were used for immunhistochemistry. This study was conducted in accordance and approval by Centralized Institutional Review Board, SingHealth Duke-NUS Academic Medical Center, Singapore. Animal studies were conducted in accordance and approval from Institutional Animal Care and Use Committee, SingHealth Duke-NUS Academic Medical Center, Singapore.

### Immunohistochemistry

Immunolocalization of KIF11/EG5 protein was performed on Validation Cohort 3 of formalin-fixed paraffin-embedded primary pediatric ATRT samples using a commercially available KIF11/Eg5 monoclonal antibody. The Leica BOND-III automated IHC Instrument (Leica Biosystems). The stained sections were reviewed by a pathologist blinded to data associated with the investigation.

### Primary tumor specimens

Fresh tumor specimens were snap frozen in liquid nitrogen and stored at − 80 °C. A total of 10 primary pediatric ATRTs collected between 1994 and 2014, and a panel of 8 patient-derived ATRT cell lines from international collaborating institutions, were extracted for RNA, used for RNA-Seq and quantitative reverse transcription polymerase chain reaction (qRT-PCR) in this study. The patient tumor samples were treatment-naïve and obtained at diagnosis, prior to treatment initiation.

### RNA-Seq, gene expression microarray and classification of SHH-ATRTs, MYC-ATRTs and TYR-ATRTs

RNA was extracted from fresh frozen patient ATRT tumor samples and a panel of 8 patient-derived ATRT cell lines using TRIzol (Invitrogen, Carlsbad, CA). Expression profiles of 10 primary human ATRTs, 8 ATRT cell lines and 10 control brain tissue RNA were generated using RNA Seq according to manufacturer’s protocol (conducted by BGI Sequencing) for the Discovery Cohort. For Validation Cohort 1, CEL files of 49 patient ATRT tumors profiled using Affymetrix HG-U133plus2.0 gene chips (Affymetrix Inc. Santa Barbara, USA), were downloaded from GSE70678^18^. Subtype identity of each patient tumor was determined by subtype-specific gene sets published^18^. Primary human ATRTs were classified into molecular subtypes, Sonic-hedgehog (SHH)-ATRTs, MYC-ATRTs and TYR-ATRTs^18^. For Validation Cohort 2, CEL files of 18 patient ATRT tumors profiled using Affymetrix HG-U133plus2.0 gene chips (Affymetrix Inc. Santa Barbara, USA), were downloaded from GSE28026^19^.

### Transcriptome sequencing (RNA-Seq)

Genome wide transcriptome analysis was performed for 28 samples (10 primary human ATRTs, 8 ATRT cell lines and 10 control brain tissue RNA) using Illumina Hiseq platform. Total RNA was extracted and treated with DNase I and mRNA were isolated using Oligo(dT) and are fragmented using the fragmentation buffer and the cDNA is synthesized. The short fragments are purified and resolved with EB buffer and added with single nucleotide A (adenine). The adapter sequences were added to the short fragments and the suitable fragments are selected for the PCR amplification. For QA/QC of the sample libraries we have used Agilent 2100 Bioanaylzer and ABI StepOnePlus Real-Time PCR System. The library is sequenced using Illumina HiSeq 4000. Paired end 2 × 100 base pair reads were generated on a HiSeq system (Illumina, San Diego, CA) and on average 8.96 Gb bases of reads was generated from each sample. The low-quality sequencing reads were removed before performing downstream analysis and the clean reads were mapped to hg19 reference genome using HISAT and on average 85.42% reads are mapped to the reference genome. After mapping sequenced reads to reference genome, the transcripts were reconstructed. A total of 23,672 coding genes were identified.

### RNA-seq data analysis

For gene expression analysis, the clean reads were mapped to reference genome using Bowtie2 (v2.2.5). Gene expression levels were calculated with RSEM (v1.2.12). To identify genes that were differentially expressed between patient tumors and normal brain controls, we performed ANOVA analysis using Partek Genome Suite. Total of 7313 genes were differentially expressed between 10 patient tumors and 10 normal brain controls with the cut-off of p-value with FDR < 0.05 and |FC > 2|. Student T-Test was used to compare KIF11 gene expression between patient tumors and control brain tissues (24.4-fold higher among patient tumors (p < 0.0001).

### Microarray data analysis

We downloaded the gene expression microarray data of 49 ATRT patient tumors from GEO (Accession ID: GSE70678^18^). Metadata containing tumor subtype identity for the 49 ATRT samples were downloaded. ATRT patient samples of three subtypes (MYC-ATRT, SHH-ATRT and TYR-ATRT) were identified previously^18^. Of 49 patient tumors, 15 tumors were MYC-subtype, 16 tumors were SHH-subtype and 18 tumors were TYR-subtype. The 49 raw CEL files were imported into Partek Genome Suite. RMA background correction and quantile normalization were performed. The gene expression values were log2 transformed and used for downstream gene expression analysis.

### Quantitative real-time polymerase chain reaction

Total RNA was extracted from cultured cells from 7 ATRT cell lines, using TRIzol Reagent (Invitrogen, US) and reverse transcription was performed using high-capacity RNA-to-cDNA kit (Applied Biosystem, US), according to manufacturer’s protocol. Real-time PCR was performed on C1000 Thermal Cycler (Bio-rad Laboratories, US) using SensiFAST Probe No-ROX kit (Bioline, UK). Each sample was loaded in triplicates. SYBR-green primers used for the reactions were *KIF11* (H_KIF11_1, Sigma-Aldrich, Germany) and housekeeping gene, *GAPDH* (H_GAPDH_1, Sigma-Aldrich, Germany). PCR was performed with initial polymerase activation at 95 °C for 2 min, followed by 40 cycles of amplification (denaturation at 95 °C for 5 s, annealing at 65 °C for 10 s and extension at 72 for 20 s). The relative expression for each mRNA was calculated by formula of ${2}^{-\Delta \Delta Ct}$.

### Proliferation assays

Tumor cells were seeded in 96-well plates in quadruplicates for each treatment condition. Ispinesib was added at the specified concentration range (CHLA-04-ATRT, CHLA-05-ATRT, CHLA-06-ATRT and BT-37 between 0.625 and 20 nM; BT-12, CHLA-266, CHLA-02-ATRT between 0.01 and 100 μM). Cell proliferation was determined using Cell Counting Kit-8. Optical density (OD) was measured using Promega GloMax-Multi Detection System.

### Flow cytometry

Tumor cells were treated in culture with ispinesib at the indicated concentrations to detect apoptosis. At each timepoint, cells were harvested and stained with Annexin V-FITC and PI according to manufacturer’s instructions (BD Annexin V FITC Apoptosis Detection Kit I, USA). Stained cells were then analyzed by flow cytometry (BD FACS Canto™ II, US) at the excitation wavelength of 488 nM (FITC) and 595 nM (PI). Cultured cells were treated with ispinesib at the indicated concentration to study the cell cycle changes. Cells were harvested and fixed with absolute ethanol. Fixed cells were stained with PI/RNase A mastermix (PI and RNase A) prior to analysis by flow cytometry (BD FACS Canto™ II, US).

### Hoechst staining

Tumor cells treated with either DMSO-vehicle or ispinesib were harvested and washed with PBS prior to fixation using 4% paraformaldehyde. Cells were stained with Hoechst 33258 (Invitrogen, US) in PBS. Slides were prepared in a pair: DMSO-vehicle treated and ispinesib-treated. Slide pairs were prepared in triplicates. Stained cells were observed under a fluorescence microscope (Nikon Eclipse 80i, US). For each slide, 20 high power fields (hpf) were captured under fluorescent microscopy. Photomicrographs of each hpf in bright field and corresponding UV field were taken. Cells with abnormal nuclear morphology (either apoptotic/condensed nuclei, Fig. S8) in 20 hpf were manually counted. Apoptotic cells were represented by a solid, clearly-demarcated rounded nucleus, while micronuclei appeared as a cluster of small, fragmented nuclei. Apoptotic cells with surrounding micronuclei were counted as micronuclei (to avoid double counting). The average number of cells per hpf was computed. The mean and standard deviations were represented on bar graphs (Fig. 5c) and the data analyzed using parametric, unpaired t-test.

### Western blotting

Protein extracts were obtained by suspending cells in RIPA buffer containing protease and phosphatase inhibitors (Sigma-Aldrich, US). Protein samples were first denatured with 5% β-mercaptoethanol in Laemmli’s buffer at 95 °C. Equal amount of denatured proteins was run on NuPAGE 4–12% Bis–Tris Protein Gel and transferred onto a membrane. The membranes were blocked with 5% milk and incubated with different primary antibodies respectively at 4 °C. Membranes were washed in TBS-T (TBS containing Tween-20) prior to incubation with respective secondary antibodies. Membranes were developed using Pierce ECL Western Blotting substrate (Thermo Scientific, US) and chemiluminescence signals were detected using ChemiDoc Imaging System, Bio-Rad Laboratories, Inc., US).

### Orthotopic implantation of patient-derived tumor cells into mouse brains

Rag2/Severe combined immune deficiency (SCID) mice were bred and housed in a pathogen-free facility. All animal experiments were conducted in accordance to protocol approved by the Institutional Animal Care and Use Committee at SingHealth Duke-NUS Academic Medical Center, Singapore. Surgical implantation of tumor cells (1 × 10^5^) into mouse cerebrum was performed using our method previously described^1,2^.

The general recommendations in ARRIVE guidelines 2.0 (https://arriveguidelines.org/) were followed in our study. Our sample size was small. We did not use any method to generate the randomisation sequence. The animals were randomly assigned into treatment versus control groups to have approximately equal number of animals per group. We have recently published that gender does not statistically affect treatment outcome^26^, and both genders were included in our study. Our animals were housed in the same location in side-by-side cages and managed in a standardized maintenance protocol by the animal unit and our team to minimize any confounding effects, we did not employ any special strategy to minimize other confounders. Treatment was delivered to animals assigned in treatment group, cage-by-cage. Blinding was not possible as we have a small team of 2–3 individuals working with the animals and we are all involved at various stages and day-to-day processes of the animal work. For ATRT tumor, the in-vivo experiment was repeated across 2 batches, and in conjunction with MB tumor, a total of 3 separate batches comprising of various litters were used, minimizing bias from the same litter effect. Additional animal data was included in our supplementary material. We have previously published using this animal protocol, and is not otherwise deposited in any repository/registry.

### Intraperitoneal drug administration

Ispinesib was dissolved in a solution of dimethyl sulfoxide, water for Batch A animals. To improve the solubility, ispinesib was dissolved in a solution of Cremophor EL, dimethyl sulfoxide, water in the subsequent Batch B animals. For both Batch A and B, ispinesib was administered intraperitoneally every 4 days for three doses, with the treatment course repeated on day 21. The dose of ispinesib was 10 mg/kg. The mice were given up to seven cycles of treatment, depending on their health fitness suitability determined by our clinical staging system. There were 9 animals (5 treated, 4 control) in Batch A and 7 animals (4 treated, 3 control) in Batch B.

### Clinical staging system to monitor mice fitness

We derived a clinical staging system to closely monitor the health fitness of mice, concurrently matching their suitability to continue on ispinesib treatment regimen (Figs. 1b,c, S3). Paralleling their human disease counterparts, some mice were physically fitter than others during the treatment course (Figs. 1b, 3a, S2–4), despite uniformly carrying the same tumor burden (Cell dose 1 × 10^5^) from orthotopically implanted ATRT cells (CHLA-06-ATRT, Fig. 1). We staged the mice from Stage 1 to 5 on a daily basis (5 days/week), using clinical categories of 8 parameters (Figs. 1c, S3) including activity, eating, grooming and grimace score^11^. Additional challenge tests (Fig. 1c) such as grip test (for strength and neurological symptoms) and mobilization test (for gait disturbance, agility) will elicit neurological signs. Mild neurological signs related to the effects of brain tumor, impairing agility, muscle weakness and gait disturbance (not affecting animal overall health fitness and function) were classified under a separate category of neurological deficits (Figs. 1c, S3A,B). Mild deficits not affecting animal overall health fitness and function were not included in the staging criteria. Progressive or generalized weakness affecting general well-being of the animals (using the 8 staging parameters) was included in the staging criteria. We targeted this window of health fitness in the mice and continued administering ispinesib to the healthier animals (between Stage 1 to 3/4, Figs. 1b, 3).

### Statistical analyses of animal survival

The survival of each mouse was taken to be the number of days from the date of tumor implantation to the date of death (overall survival) or date of progression to Stage 3 or 4, where event = 1 and censored subject = 0. Data were censored for mice that were alive at the time of analysis. All Kaplan–Meier survival curves were plotted using GraphPad Prism 8.1.0 and the survival analysis was performed using Log-rank (Mantel-Cox) test.

## Supplementary Information

Supplementary Information.

## Data Availability

The Cancer Genome Atlas (TCGA) Research Network; Accession codes-GSE42670, GSE19578, GSE74187. Non-commercial cell lines and materials used in this project were obtained through an MTA with international collaborating institutions.
