# Supplementary material for: Maximizing the potential of aggressive mouse tumor models in preclinical drug testing
Source: Sci Rep. 2021 Jun 2;11:11580. doi: 10.1038/s41598-021-91167-6 (PMC8172610; doi:10.1038/s41598-021-91167-6)

Maximizing the potential of aggressive mouse tumor models in preclinical drug testing

Authors

M. Tarek Elghetany[1,2], Jia-Min Ho[3,4], Lois Hew Shi-Qi[3,4], Sekar Karthik[3], Jack MF Su[1,5,6], Qi Lin[7,8], YuChen Du[7,8], Jianhe Shen[1,5], Wing-Yuk Chow[1,5], Ching C Lau[1,5,6,9], Adekunle Adesina[1,6,10], Angela Major[1,2], Anat Erdreich-Epstein[11], Kam-Man Hui[3,12,13], Xiao-Nan Li[1,5-8], Wan-Yee Teo[1,3-6,12-14]*

Affiliations

[1]Baylor College of Medicine, Houston, TX, USA.
[2]Department of Pathology, Texas Children's Hospital, Houston, TX, USA.
[3]Humphrey Oei Institute of Cancer Research, National Cancer Center Singapore.
[4]Pediatric Brain Tumor Research Office, SingHealth-Duke-NUS Academic Medical Center, Singapore.
[5]Department of Pediatrics, Division of Hematology-Oncology, Texas Children's Cancer Center, USA.
[6]Dan L. Duncan Cancer Center, Houston, TX, USA.
[7]Ann & Robert H. Lurie Children's Hospital of Chicago, IL, USA.
[8]Northwestern University Feinberg School of Medicine, IL, USA.
[9]Connecticut Children's Medical Center, The Jackson Laboratory for Genomic Medicine, University of Connecticut School of Medicine, USA.
[10]Department of Molecular Pathology, Texas Children's Hospital, Houston, TX, USA.
[11]Departments of Pediatrics and Pathology, Children's Hospital Los Angeles, Norris Comprehensive Cancer Center, and the Keck School of Medicine, University of Southern California, USA
[12]Institute of Molecular and Cell Biology, A*STAR, Singapore.
[13]Cancer and Stem Cell Biology Program, Duke-NUS Medical School, Singapore.
[14]KK Women's & Children's Hospital, Singapore.

***Corresponding Author:** Assistant Professor Wan-Yee Teo, *MBBS, FAAP, MRCPCH (UK), FRCP Edin, FAMS, PhD*
Correspondence to wan-yee.teo@duke-nus.edu.sg

Supplementary Materials

Fig. S1

A. Establishment of a large panel of patient-derived orthotopic xenograft (PDOX) mouse models using patient tumors and patient-tumor-derived cell lines of ATRT. CHLA-06 was the most aggressive phenotype in our panel of ATRT tumor models.

A.

ATRT Tumor Cell Lines	Xenograft Tumor Implantation	Tumor Formation	Survival Characteristics	Median Survival Days	Number of Mice
CHLA-02	Yes	No	2 early animal deaths (Day 4, Day 21) likely non-tumor related	197 days	1
CHLA-04	Yes	No	NA	243 days	5
CHLA-05	Yes	Yes	NA	151 days	4
CHLA-06	Yes	Yes	Highly aggressive. Invariably lethal in all animals within 1 month of tumor implantation	22 days	14
CHLA-266	Yes	No	NA	235 days	5
BT-12	Yes	No	NA	77 days	5
BT-37	Yes	Yes	Median survival longer than CHLA-06 model	63.5 days	4
ATRT95	No	NA	NA	NA	NA
**ATRT Patient Tumors**	**Xenograft Tumor Implantation**	**Tumor Formation**	**Survival Characteristics**		
ICb-10593ATRT	Yes	Yes	Data not shown; Median survival longer than CHLA-06 model		
IC-1218ATRT	Yes	Yes	Data not shown; Median survival longer than CHLA-06 model		
ICb-1355ATRT	Yes	Yes	Data not shown; Median survival longer than CHLA-06 model		
ICb-5108ATRT	Yes	Yes	Data not shown; Median survival longer than CHLA-06 model		
ICb-L1115ATRT-CSC	Yes	Yes	Data not shown; Median survival longer than CHLA-06 model		

Fig. S1

B. Survival curves of different PDOX tumor models studied. *Upper Panel:* Survival curves of multiple cohorts (different experimental batches) of PDOX mouse models bearing CHLA-06 tumor cells (Cell dose 1X10^{-5}) demonstrating uniform aggressive behaviour of this ATRT tumor model. This tumor model was invariably lethal in all mice within ~1 month of tumor implantation across multiple, reproducible cohorts of mouse xenografts. *Lower Panel:* Survival curves of other PDOX tumor models created with various patient-derived ATRT cell lines using the same orthotopic implantation technique (see ***Methods***).

B. **Survival curves for multiple batches of CHLA-06**

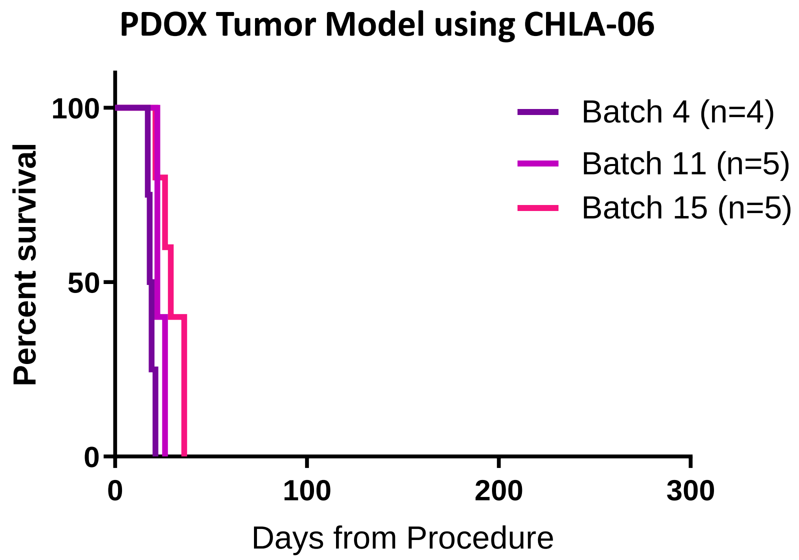

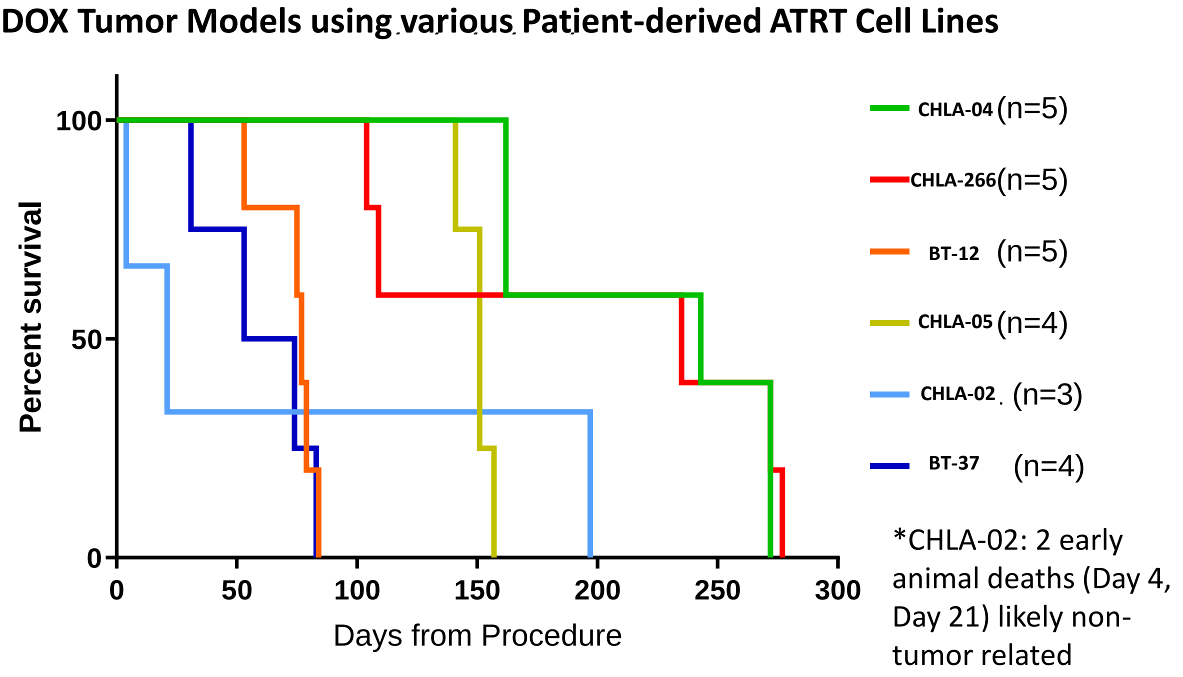

Fig. S2

A. Schematic illustration of our Fitness-adapted approach to preclinical drug testing extracted from **Fig. 1b**.

B. Table showing treatment days of ispinesib in *Batch A* animals (total 2 cycles were delivered), and *Batch B* animals (up to 7 cycles were delivered). Our clinical staging system was used to evaluate the health fitness of the mice daily (5 days/week) to determine further cycles of ispinesib. Ispinesib *in-vivo* dosing is 10mg/kg administered intraperitoneally every 4 days for three doses, with the treatment course repeated around day 21, depending on the mice fitness level. At Stage 3-4, we would monitor the health of the mice over the next few days before deciding if the mice remain stable/fit to receive additional cycles.

A.

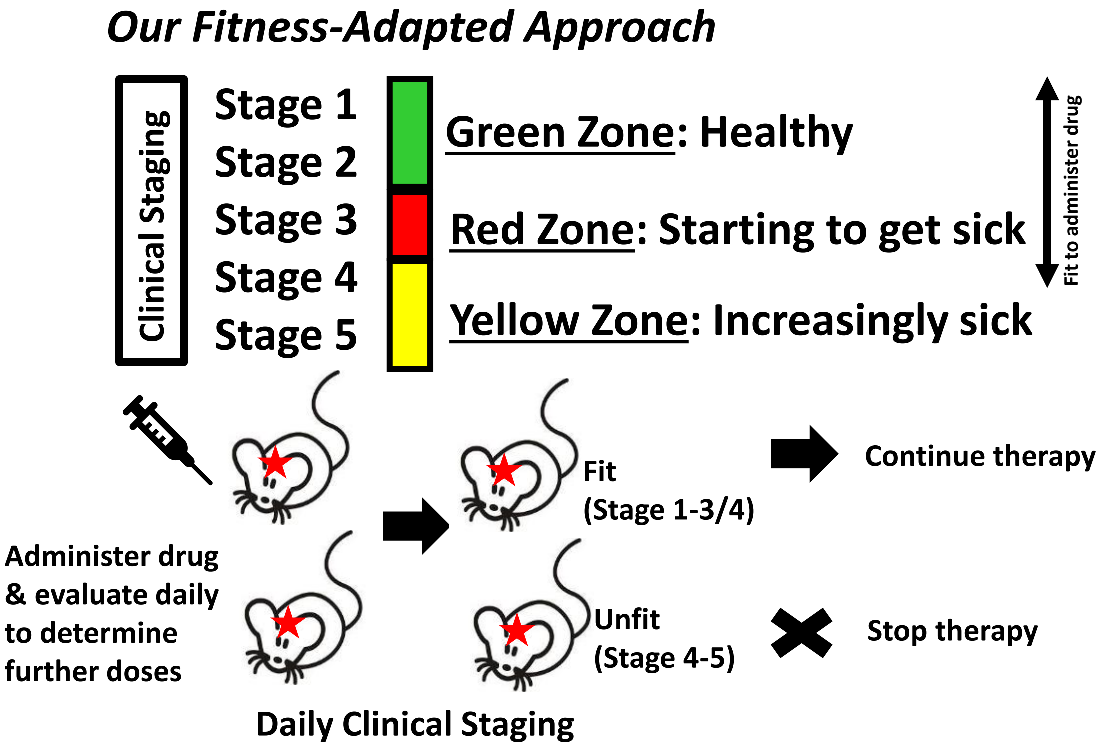

B. Cycles of Drugs Delivered

Batch A	Treatment Dates (Day post tumor implantation)	Number of Days post tumor implantation at start of treatment	Number of Mice Treated	Number of Mice at End of Cycle
**1st Cycle**	13d, 17d, 22d	13	5	2
**2nd Cycle**	34d, 38d, 42d	34	2	0

Batch B	Treatment Dates (Day post tumor implantation)	Number of Days post tumor implantation at start of treatment	Number of Mice Treated	Number of Mice at End of Cycle
**1st Cycle**	15d, 19d, 23d	15	4	3
**2nd Cycle**	36d, 40d, 44d	36	3	3
**3rd Cycle**	57d, 61d, 65d	57	3	2
**4th Cycle**	75d, 79d, 83d	75	2	2
**5th Cycle**	96d, 100d, 104d	96	2	1
**6th Cycle**	120d, 124d, 128d	120	1	1
**7th Cycle**	141d, 145d, 149d	141	1	1

Fig. S3

A. Illustrated staging table on how the animals are staged. Note that not every animal displays the same pattern of progression in each parameter. The higher stage was used.

B. Pictures demonstrating how additional challenge test (grip test) is performed. Mice were held at this angle to perform observations on the hind legs as well as the front legs, to determine how the mouse was moving its limbs in a daily examination. Holding the mouse too near the base of its tail will cause the examining hand to obstruct the view of the hind leg movements. This grip test manoeuvre allowed us to observe the mouse doing a front grip onto the grills, and back-kick on the hind legs.

A.

STAGING TABLE	Stage 1	Stage 2	Stage 3	Stage 4	Stage 5 (Moribund)
**Activeness/ Movement in cage**	Actively runs about		Runs around, can have occasional stagger.<br>Less engaged with other mice	Interested in surroundings, but less active/engaged with other mice	Refuses to respond
**Reaction when picked up**	Struggles strongly		Struggles fairly strongly	Struggles less strongly	Does not struggle or resist
**Grip strength (Generalized weakness)**	Strong		Fairly strong/Strong	Less strong grip	Very weak/does not grip
**Body posture**	Normal	Slight head bulge and/or mildly hunched back	Head bulge. Mildly hunched back	Prominent head bulge. Mildly/ hunched back	Lying down
**Body temperature**	Normal				Low body temperature. Shivering
**Appetite**	Normal			Eating but may occasionally drop the food	Not interested in eating
**Grooming**	Well-groomed		May have messy fur		
**Grimace Score[10]**	Normal		Slight nose/cheek bulge, orbital tightening leading to narrowed eyes	Relatively more prominent nose/cheek bulge, orbital tightening leading to narrowed eyes	

B.

How Grip Test is Performed

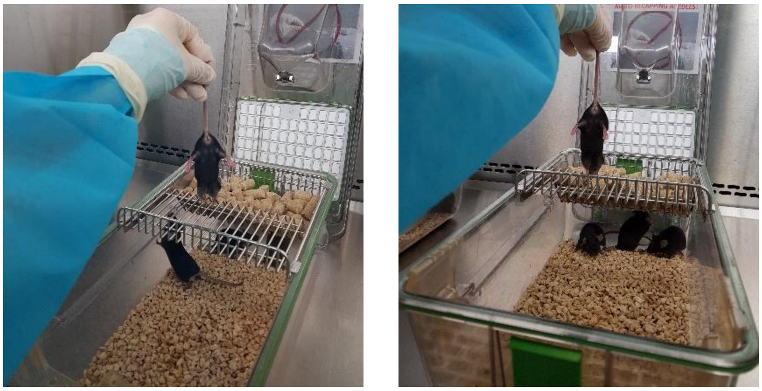

Fig. S4

A. Table showing the day of first symptoms observed, fulfilling Stage 3, 4 and 5 for *Batch A* animals. Stage 1 and 2 mice were in optimal health state. This data was used to analyse progression-free and overall survival as shown in **Fig. 1h, i**.

A.

Batch A animals

Mouse ID	Date of First Symptom (Days after tumor implantation)	Date of Stage 3	Date of Stage 4	Date of Stage 5	Date of Death (Days after tumor implantation)	Dates of Treatment Cycle Initiated (Each Cycle comprised of 3 doses-Dose #1, Dose #2, Dose #3)
**Untreated**						
#1197	25d	25d	27d	-	28d Died	
#1302	27d	27d	29d	-	29d Sacrificed	
#1194 (1311)	22d	25d	25d	25d	25d Died	
#1195	22d	25d	25d	25d	25d Died	
**Ispinesib-Treated (1 Cycle)**						
#1198	29d	34d	34d	36d	36d Sacrificed	
#1193	22d	28d	29d	-	30d Sacrificed	
#1196	22d	27d	29d	-	30d Sacrificed	
**Ispinesib-Treated (2 Cycles)**						
#1200	27d	27d	38d	-	50d Died	13d, 17d, 22d<br>34d, 38d, 42d
#1301	27d	27d	53d	-	54d Died	

Fig. S4

B. Table showing the day of first symptoms observed, fulfilling Stage 3, 4 and 5 for *Batch B* animals. Stage 1 and 2 mice were in optimal health state. This data was used to analyse progression-free and overall survival as shown in **Fig. 1h, i**.

B.

Batch B animals

Mouse ID	Date of First Symptom (Days after tumor implantation)	Date of Stage 3	Date of Stage 4	Date of Stage 5	Date of Death (Days after tumor implantation)
**Untreated**					
#1203	29d	33d	33d	-	33d Sacrificed
#1205	26d	26d	28d	-	28d Sacrificed
#1207	26d	26d	26d	-	26d Sacrificed
**Ispinesib-Treated (1 Cycle)**					
#1209	29d	36d	36d	-	36d Sacrificed
**Ispinesib-Treated (3 Cycles)**					
#1204	44d	48d	68d	-	69d Sacrificed
**Ispinesib-Treated (5 Cycles)**					
#1206/1222	99d	110d	113d	-	113d Sacrificed
**Ispinesib-Treated (7 Cycles)**					
#1208/1223	114d	152d	187d	-	187d Sacrificed

Fig. S5

A. Top view of cranial cavity showing mice brains in *Batch A* animals (Treatment Group=5, Control Group=4). Brains from ispinesib-treated animals (*lower 2 panels*) were more normal in appearance, in contrast to untreated control animals (*upper panel*) which were more necrotic in appearance. Similar findings were observed in *Batch B* animals (**Fig. 1g**).

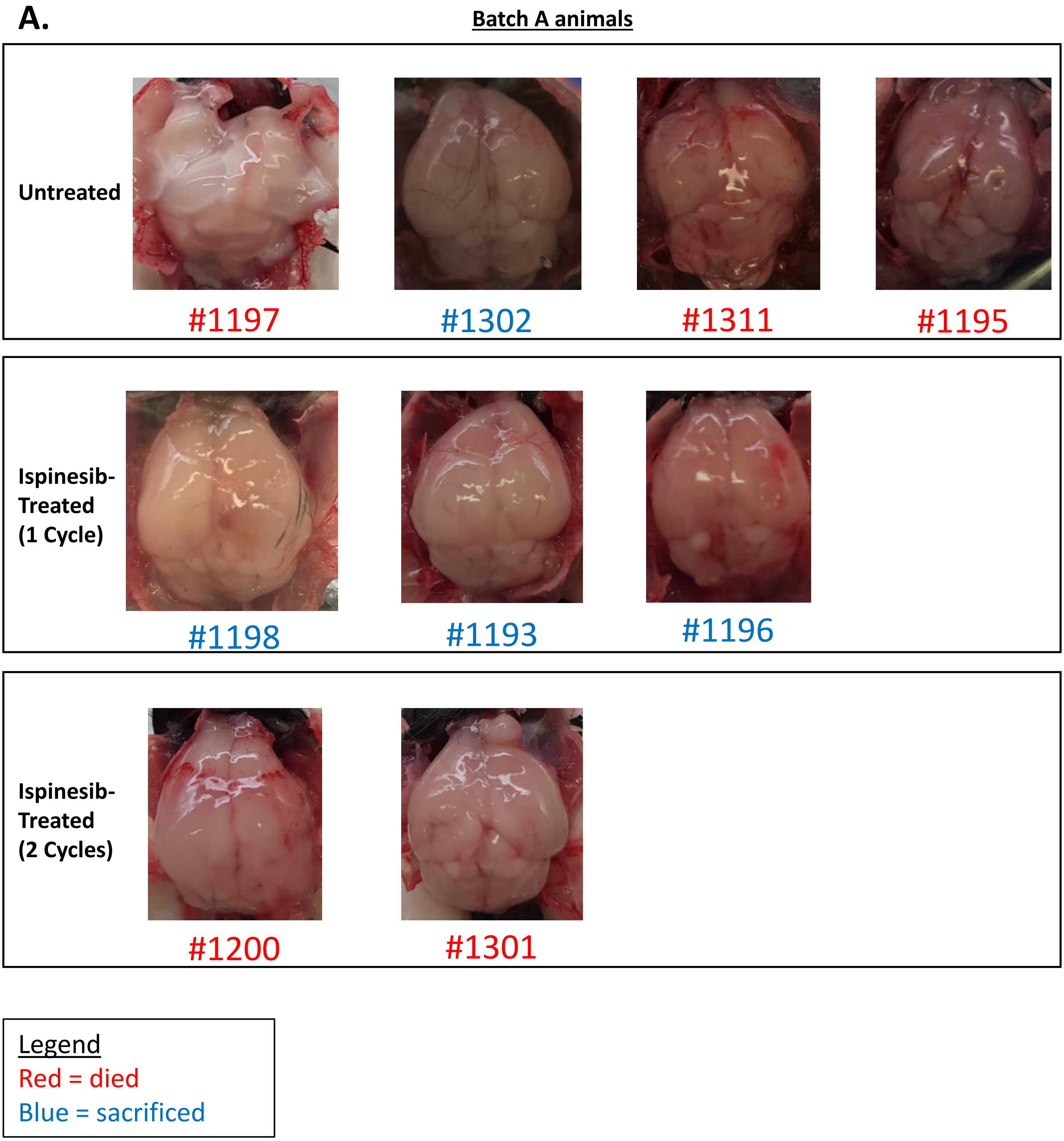

Fig. S5

B. Hematoxylin & eosin staining of each corresponding mouse brain sectioned in *Batch A* animals. ***Red*** *arrows* indicate tumor formation. Some brain specimens have poor quality due to overnight deaths of animals (#1197, #1311, #1200, #1301). #1301 did not appear to have any visible residual tumor, area indicated by ***red*** *arrow* demonstrated no tumor cells but a cavitation.

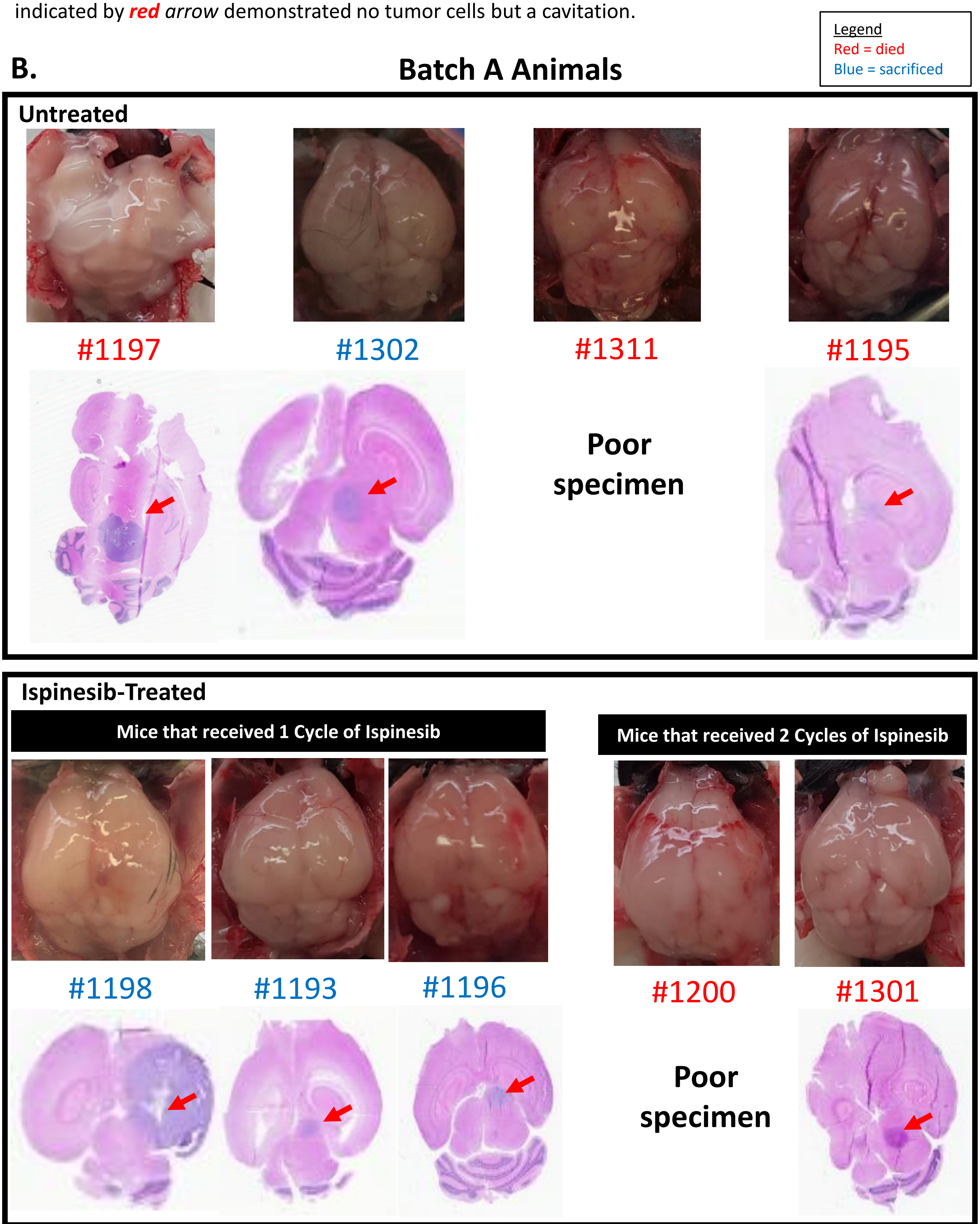

Fig. S6

A. KIF11 gene expression in relation to GAPDH (housekeeping gene) in patient ATRT tumors of 3 molecular subtypes in Validation Cohort 1 (n=49, Microarray Affymetrix U133plus2.0, GSE70678[18]).

B. Immunohistochemistry demonstrating KIF11 protein expression on (CHLA-06 xenografts and) patient ATRT tumors (Validation Cohort 3). Patient 1's tumor with approximately 10-20% staining (***red*** *arrows* indicate mitoses, ***green*** *arrows* indicate apoptotic bodies). KIF11 protein expression co-localized with mitoses. Patient 2's tumor with approximately 5% positive cells. Patient 3's tumor with 20-30% positive cells.

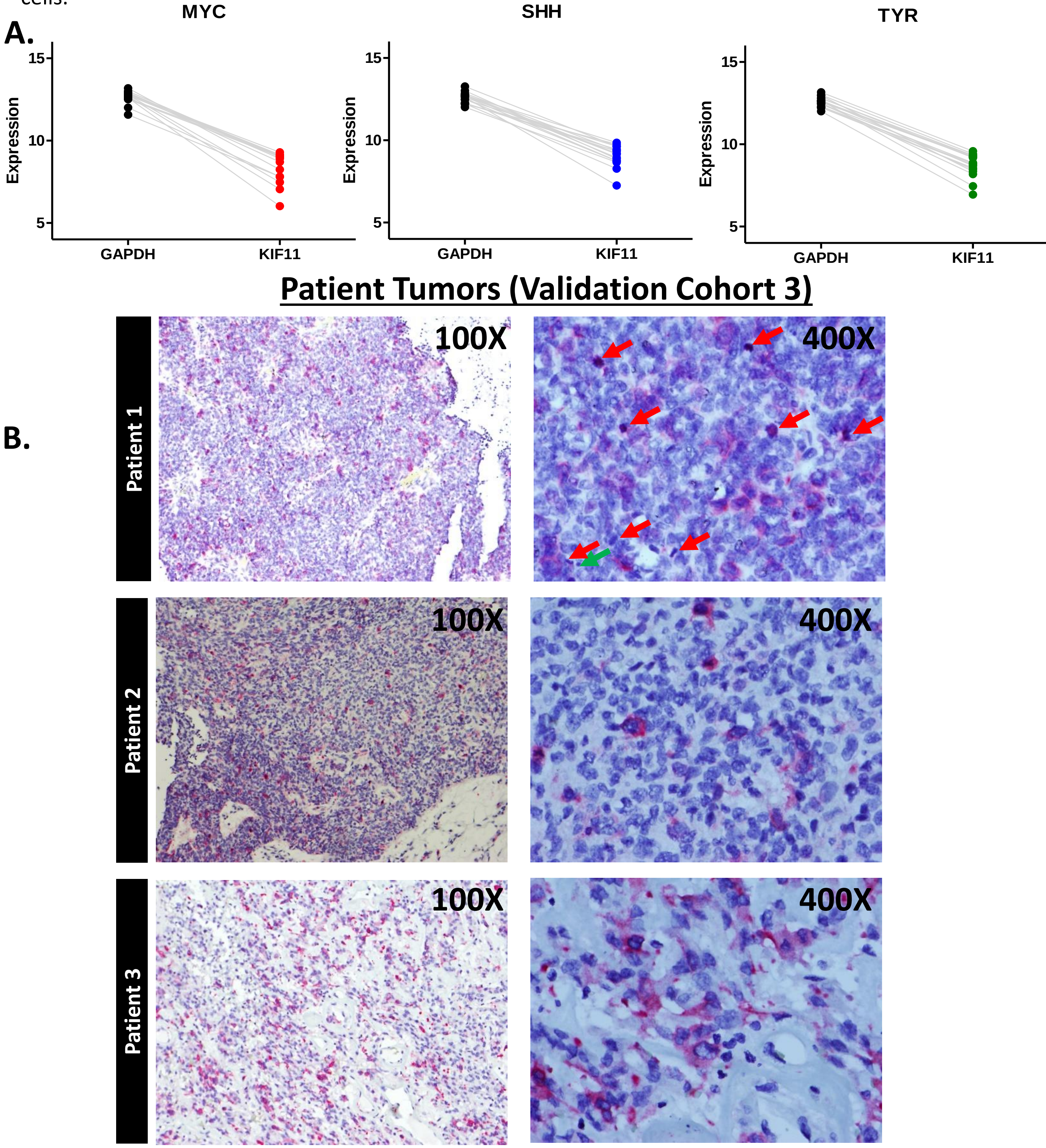

Fig. S6

C. Western blot showing KIF11 protein expression across 7 ATRT cell lines (experimental replicate to **Fig. 4d)**. Single blot, *black line* delineating section cropped as sample was suboptimal.

D. qRT-PCR replicate showing KIF11 mRNA expression across 7 ATRT cell lines (experimental replicate to **Fig. 3a**).

E. Western blot showing increase in apoptotic markers (c-PARP, c-caspase 3) and DNA damage marker (p-H2AX) with ispinesib treated CHLA-05 ATRT cells, CHLA-06 ATRT cells (replicate of **Fig. 4d)** and BT-37 ATRT cells (replicate of **Fig. 4d)** .

Replicate

C.

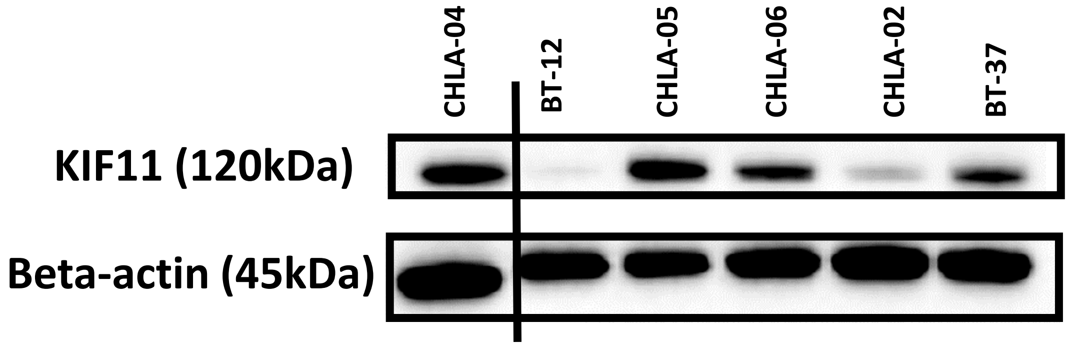

D. **Replicate 1**

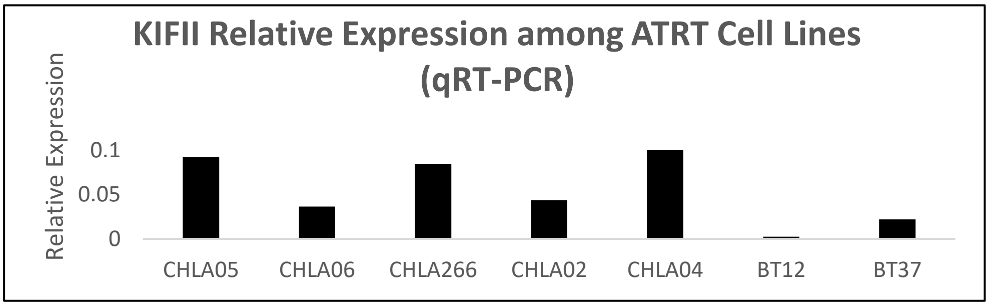

*2^-(ΔCt) = Relative Expression

E.

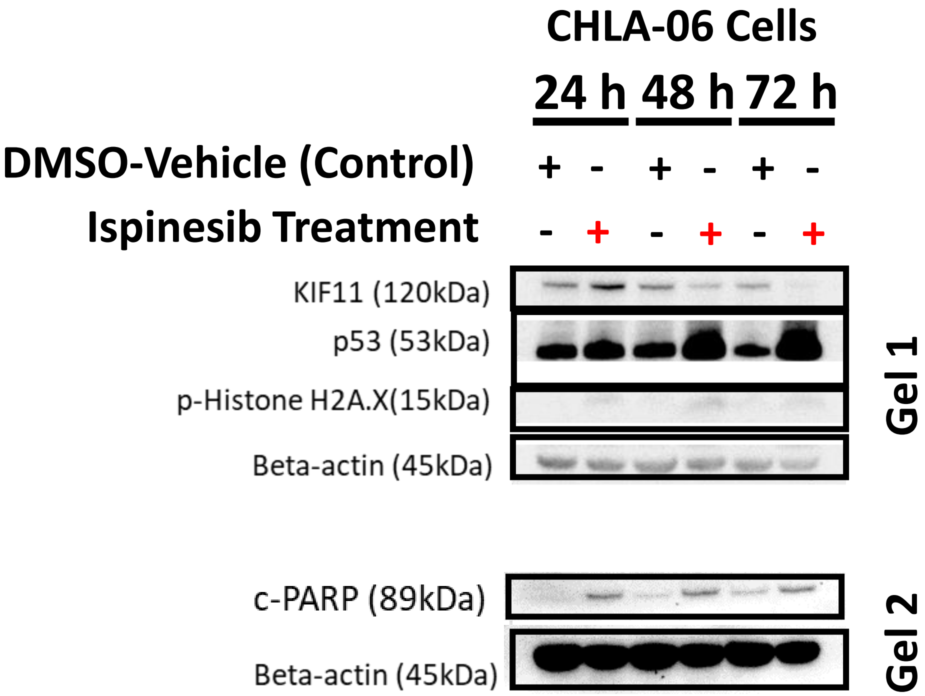

Fig. S6
Continued
E.

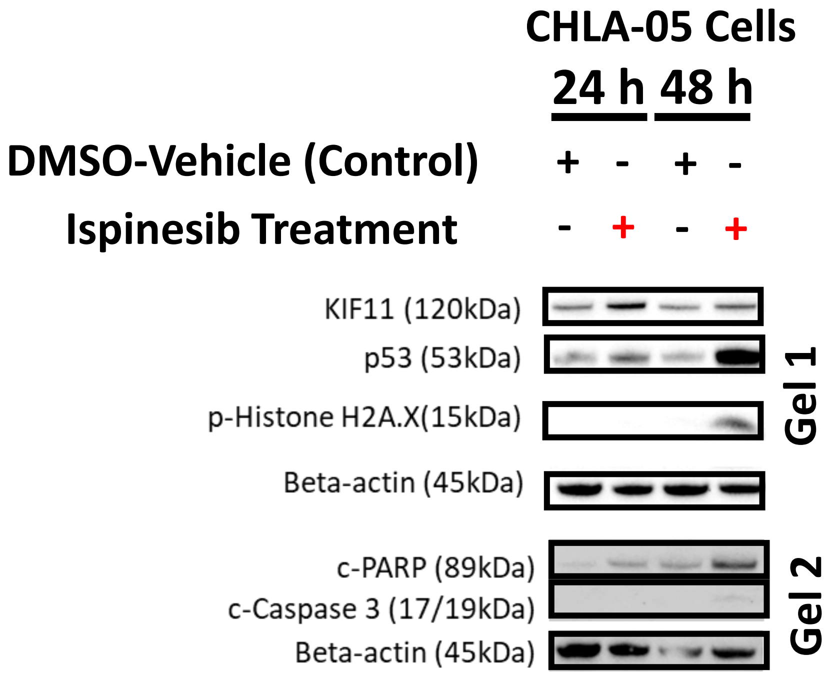

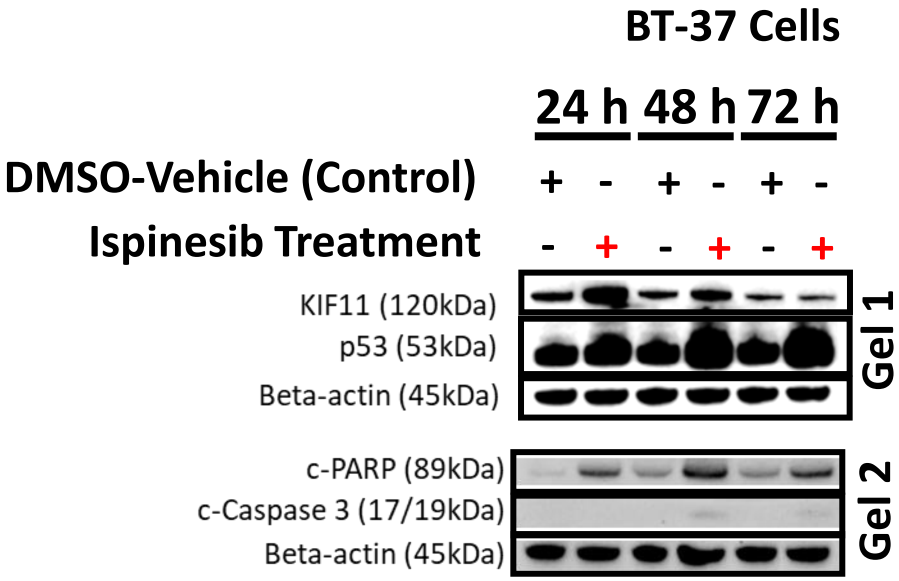

Fig. S7

A. Additional ATRT cell lines (CHLA-05) were treated with ispinesib and evaluated for cell cycle changes (**Fig. S7A**) and apoptosis (**Fig. S7B**). Cell cycle analysis of CHLA-05 treated with ispinesib (17.7 nM) for 24 h by propidium iodide flow cytometry. CHLA-05 cells treated with ispinesib showed increased percentage in G2/M phase by 7.4% as compared to DMSO control. This increase was accompanied by the concomitant decrease in G1 phase by 19% and increase in sub-G1 phase by 11.8%. This result indicated that ispinesib induced G2/M arrest and subsequent cell death in CHLA-05 cells.

A.

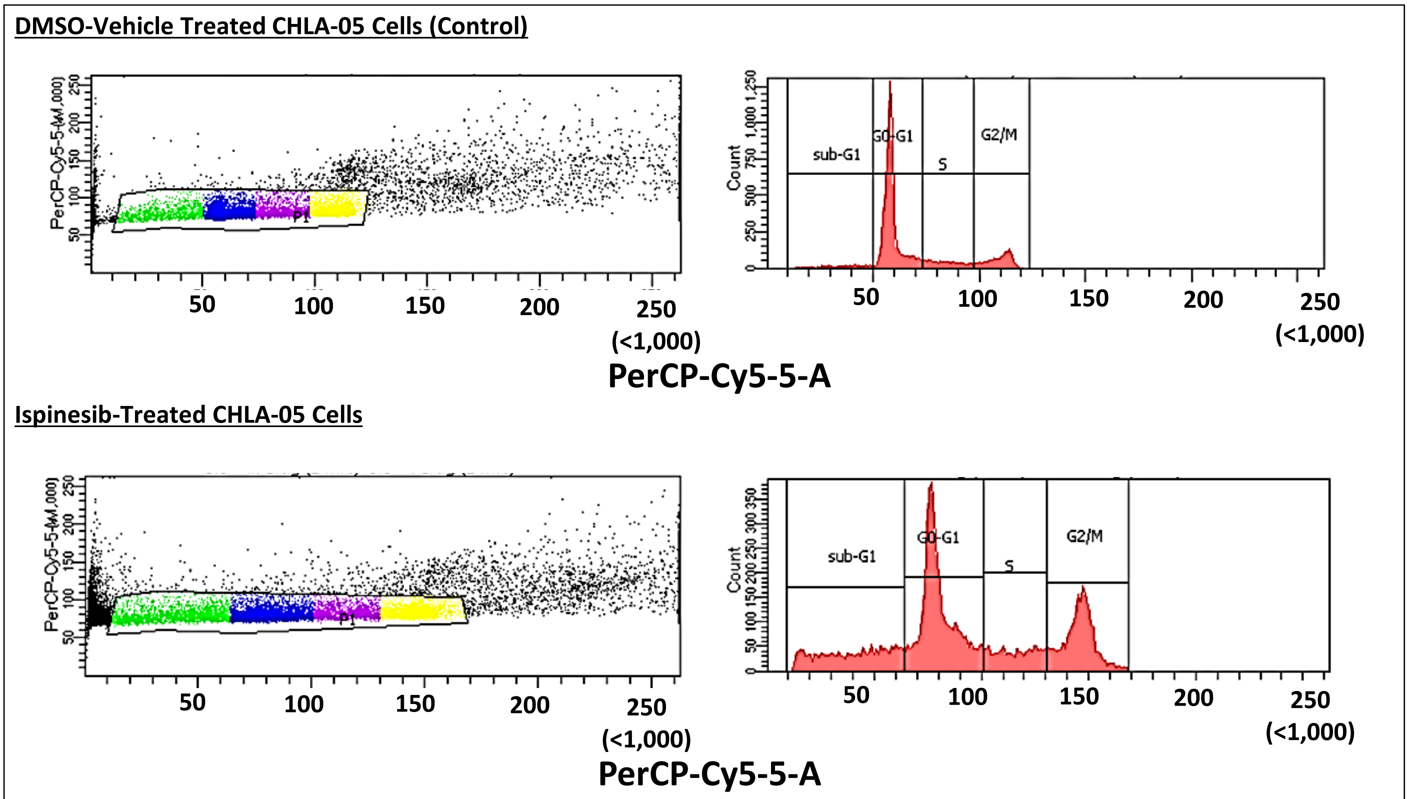

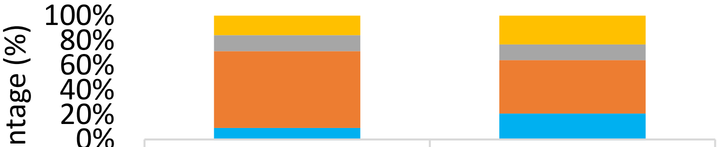

	DMSO control	I drug treated
G2/M	15.8	23.2
S	12.9	12.8
G1	62	43
Sub-G1	9.3	21

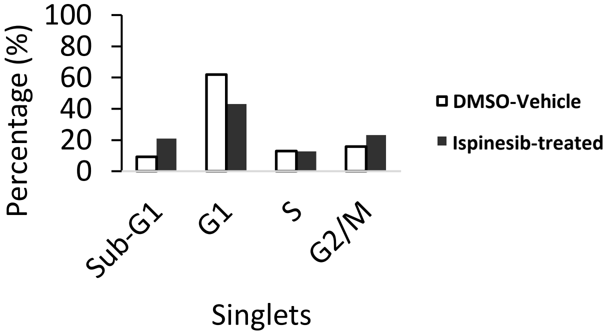

Fig. S7

B. Corresponding CHLA-05 cells were treated with ispinesib and evaluated for cell death and apoptotic changes. Detection of apoptosis marker in CHLA-05 cells treated with ispinesib (17.7 nM) for 24 and 48 h. Percentage of secondary necrotic cells (AnV^+/PI^+ cells) was higher after 48 hrs (23.5%). There was no difference in apoptotic cells (AnV^+ only) between DMSO control and ispinesib-treated cells.

B.

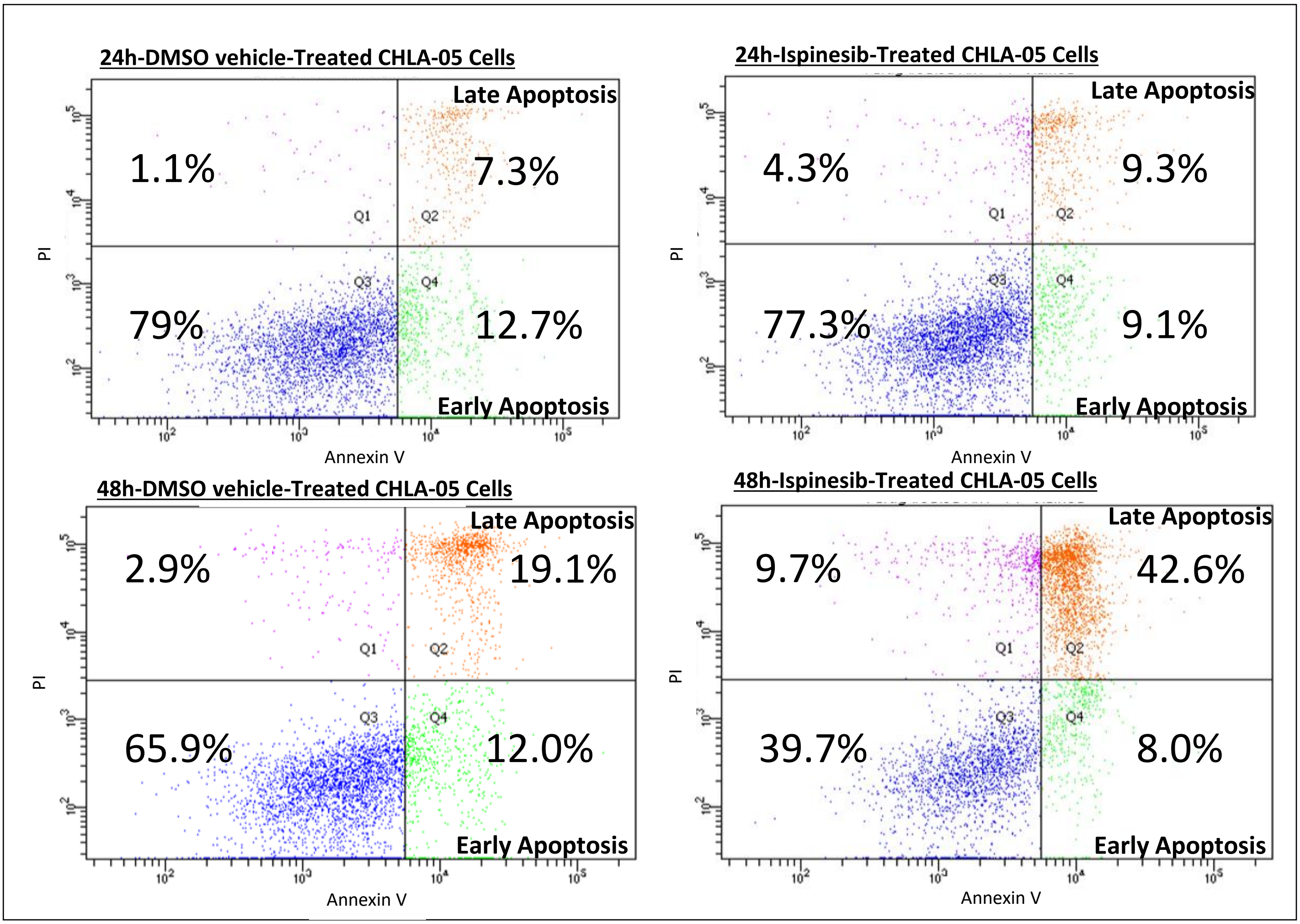

Fig. S7

C. KIF11 is a universal target across all ATRT-subtypes and KIF11-targeting with ispinesib inhibited proliferation in a panel of 7 ATRT cell lines. IC_{50} curves of ispinesib on all 7 cell lines (Day 7). Time-dependent effects of ispinesib on all 7 ATRT cell lines by ispinesib (lowest effective dose for each cell line) over 13 days.

C.

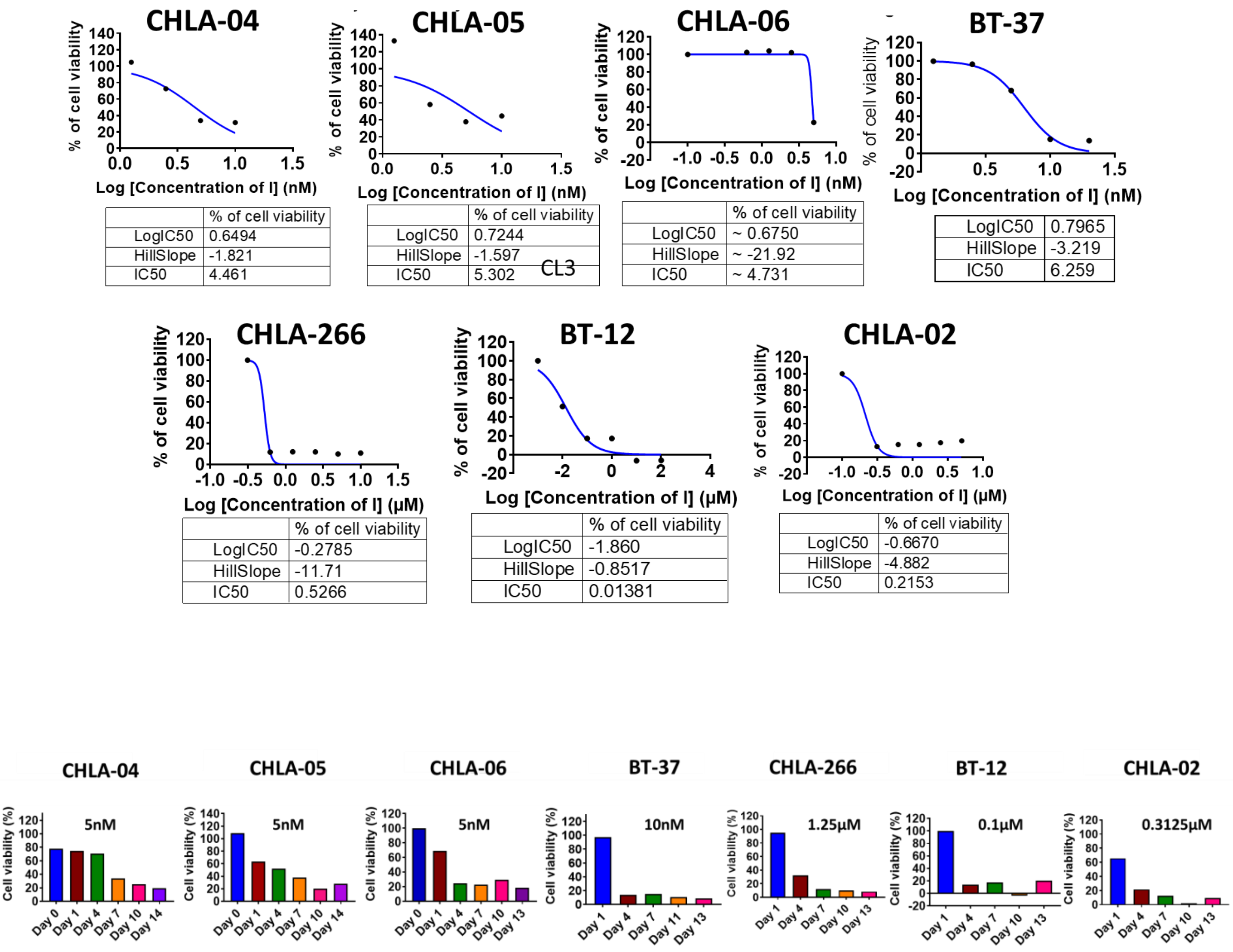

Fig. S8

A. Nuclear phenotypes observed in ispinesib-treated BT-37 cells (10nM ispinesib).

B. Nuclear phenotypes observed in ispinesib-treated CHLA-06 cells (4.69nM ispinesib).

A.

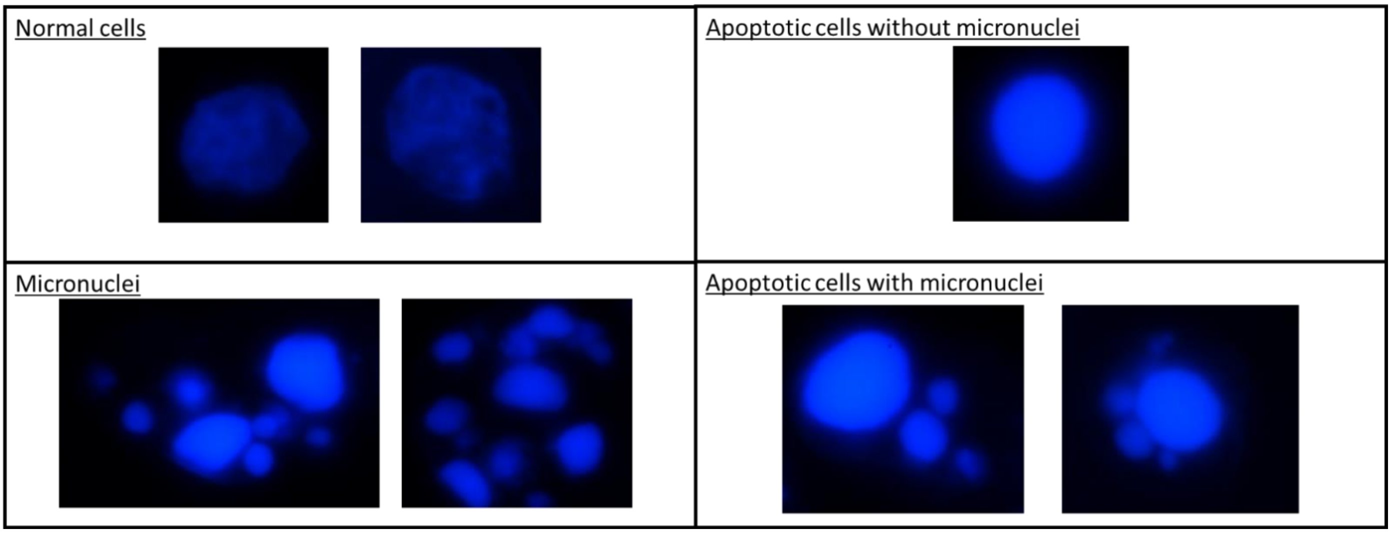

B.

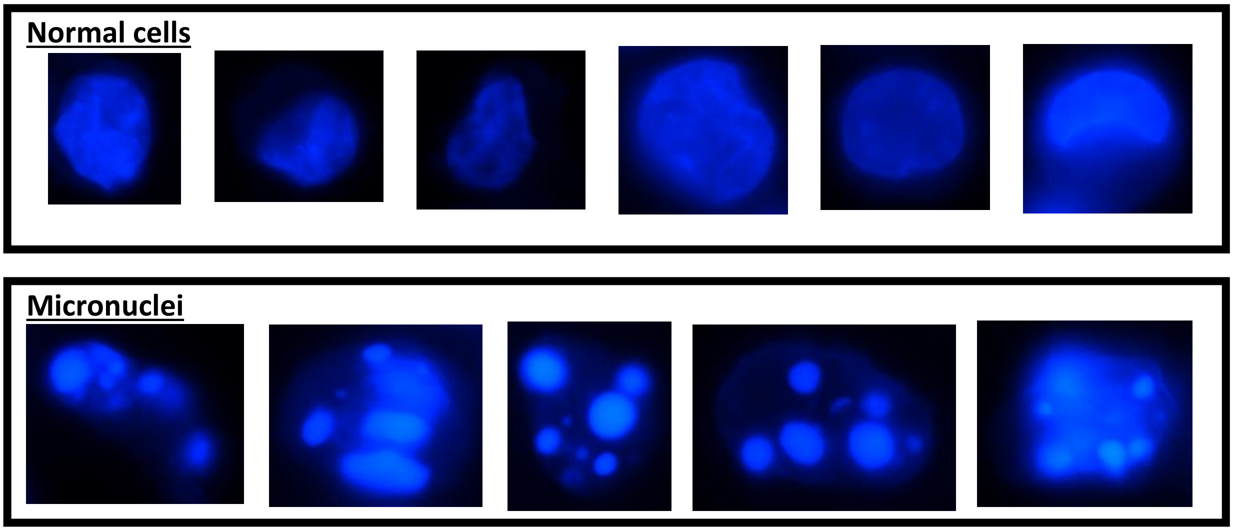

Fig. S9

Schematic

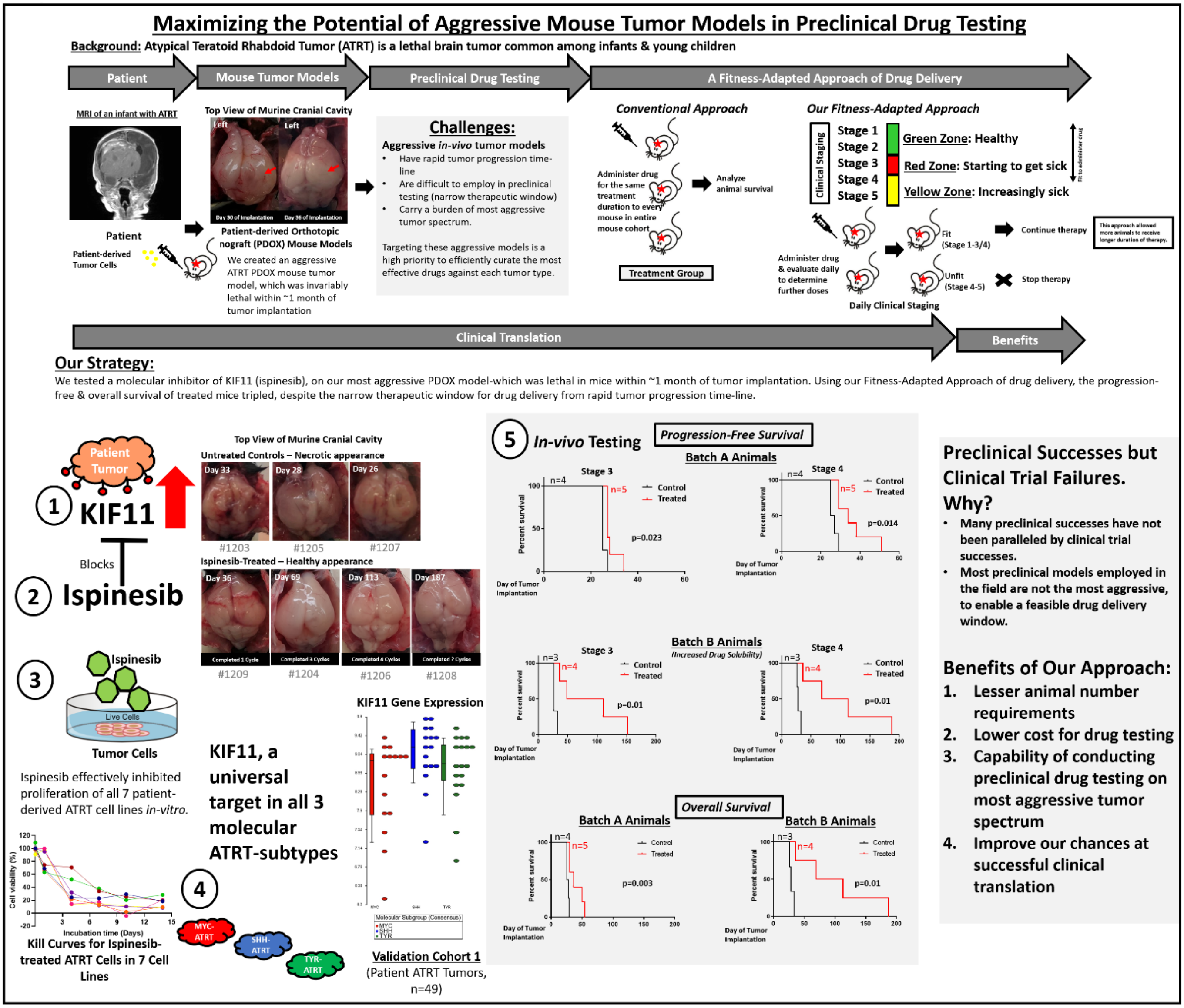

Fig. S10

Full length western blots for western blots used in main figures (**Fig. 4d**). Consistent brightness for each gel.

Consistent Brightness

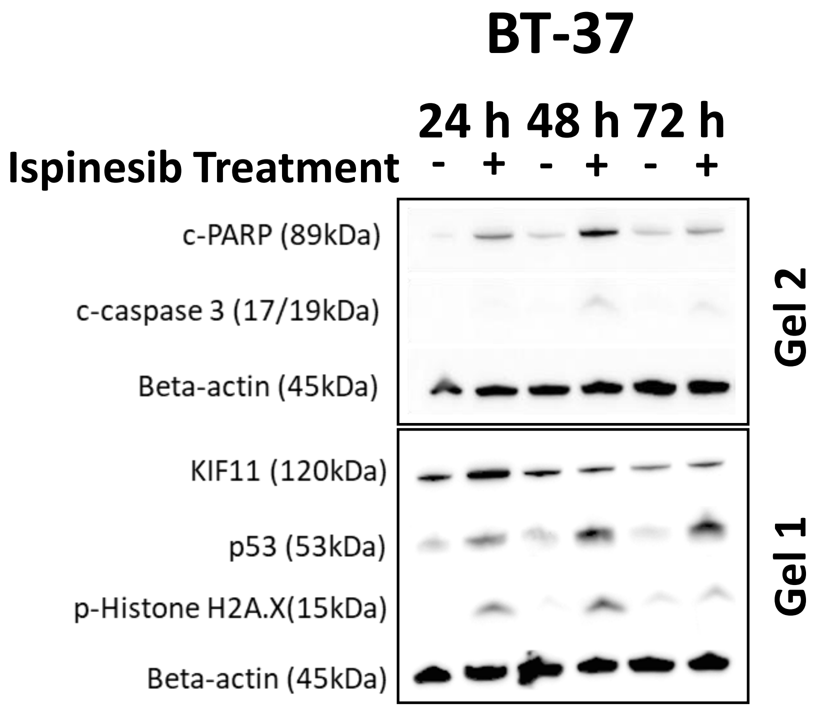

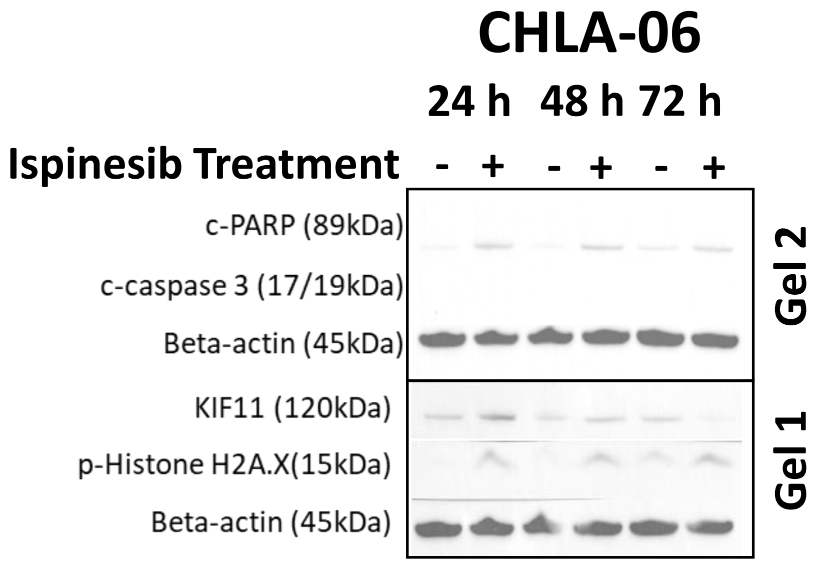

Full Length Blot (without ladder)

Original full length blots for **Fig. 4d.** Aspect ratios were not altered and the cropped images shown in the main figures and their corresponding originals were taken at the same exposure level. Only on Microsoft powerpoint settings, the brightness was adjusted for the main figure clarity.

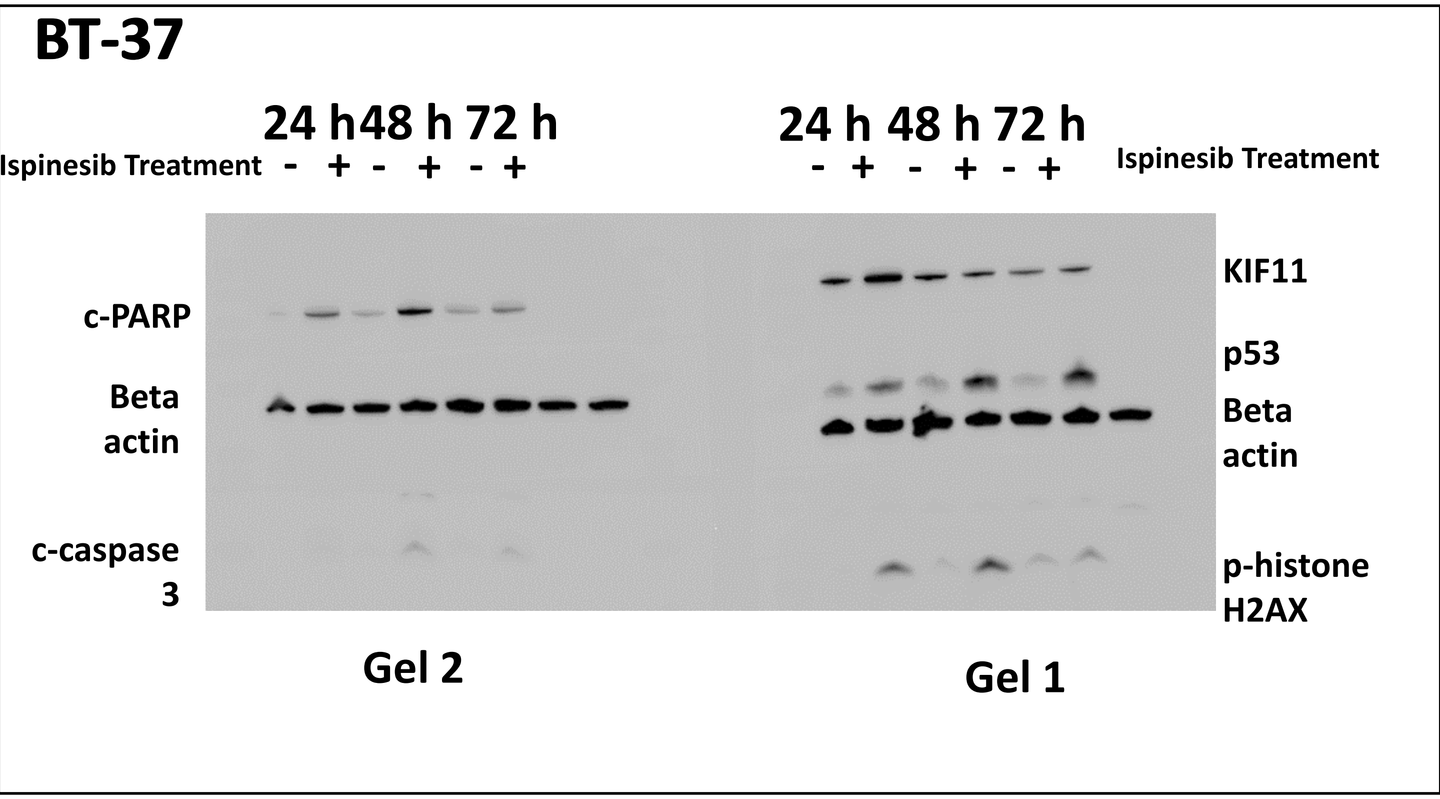

Full Length Blots (with ladder)

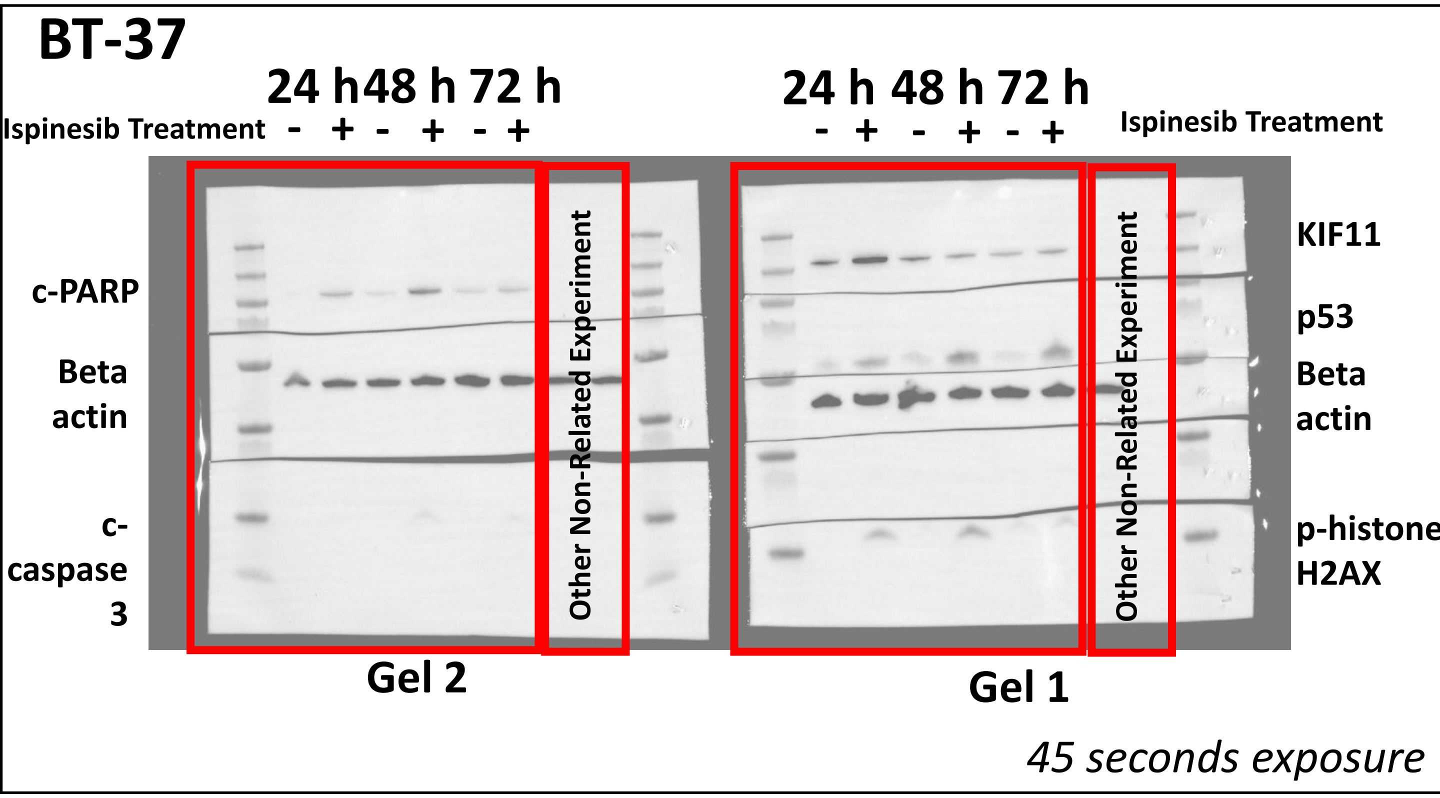

Full Length Blots

Original full length blots for **Fig. 4d.** Aspect ratios were not altered and the cropped images shown in the main figures and their corresponding originals were taken at the same exposure level. Only on Microsoft powerpoint settings, the brightness was adjusted for the main figure clarity.

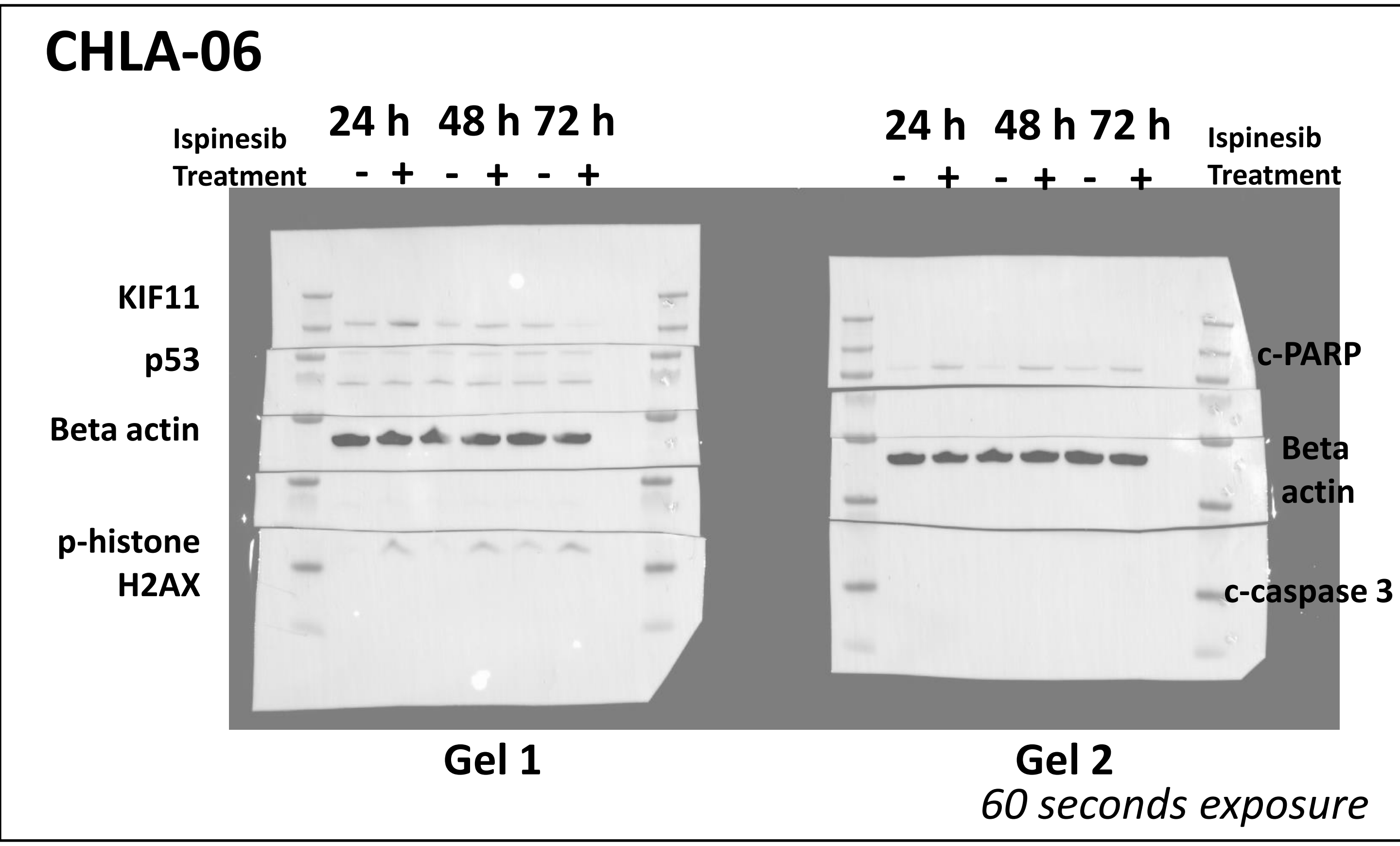

Full Length Blots

Original full length blot for **Fig. 4d.**

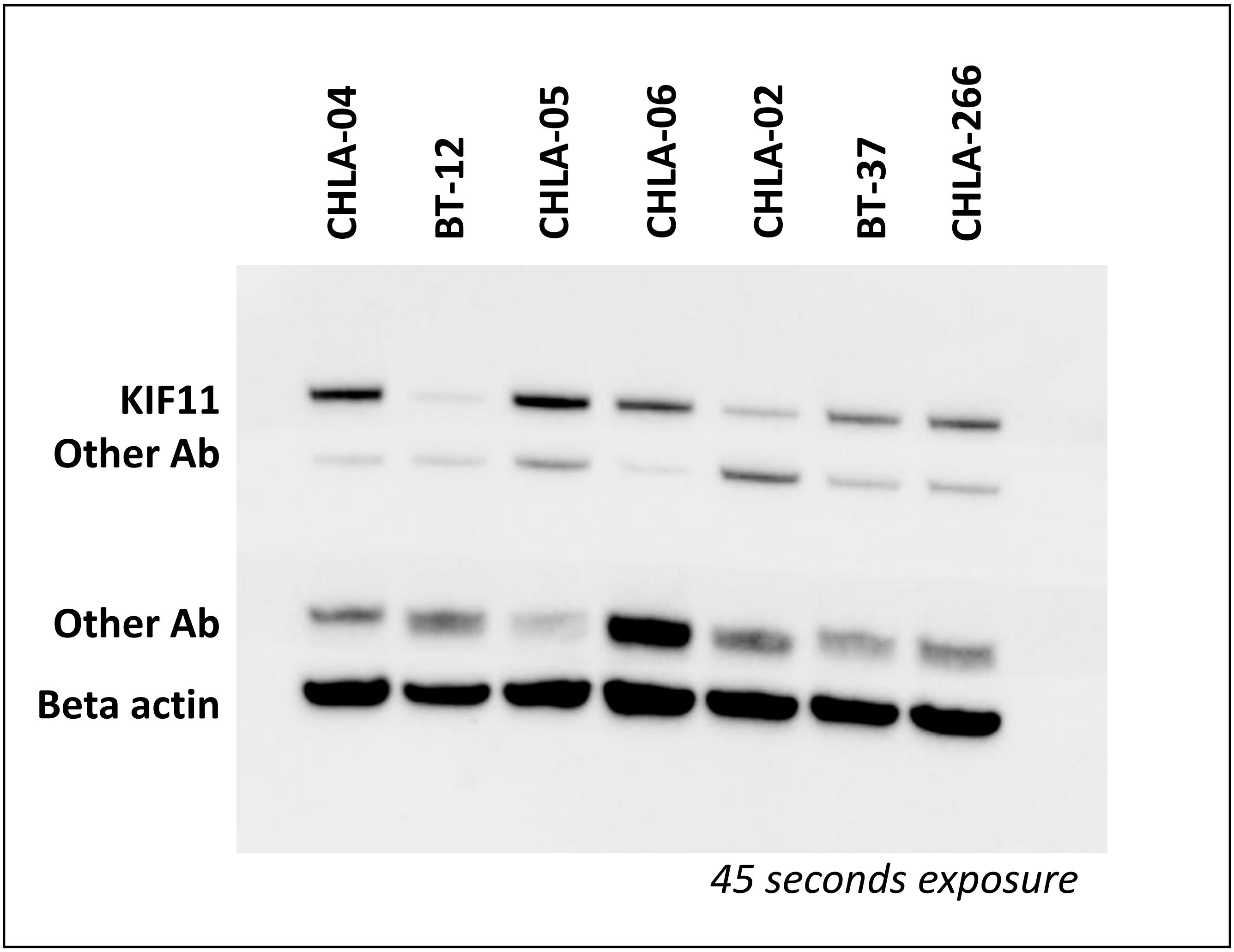

Full Length Blots

Original full length blot for **Fig. S6C.**

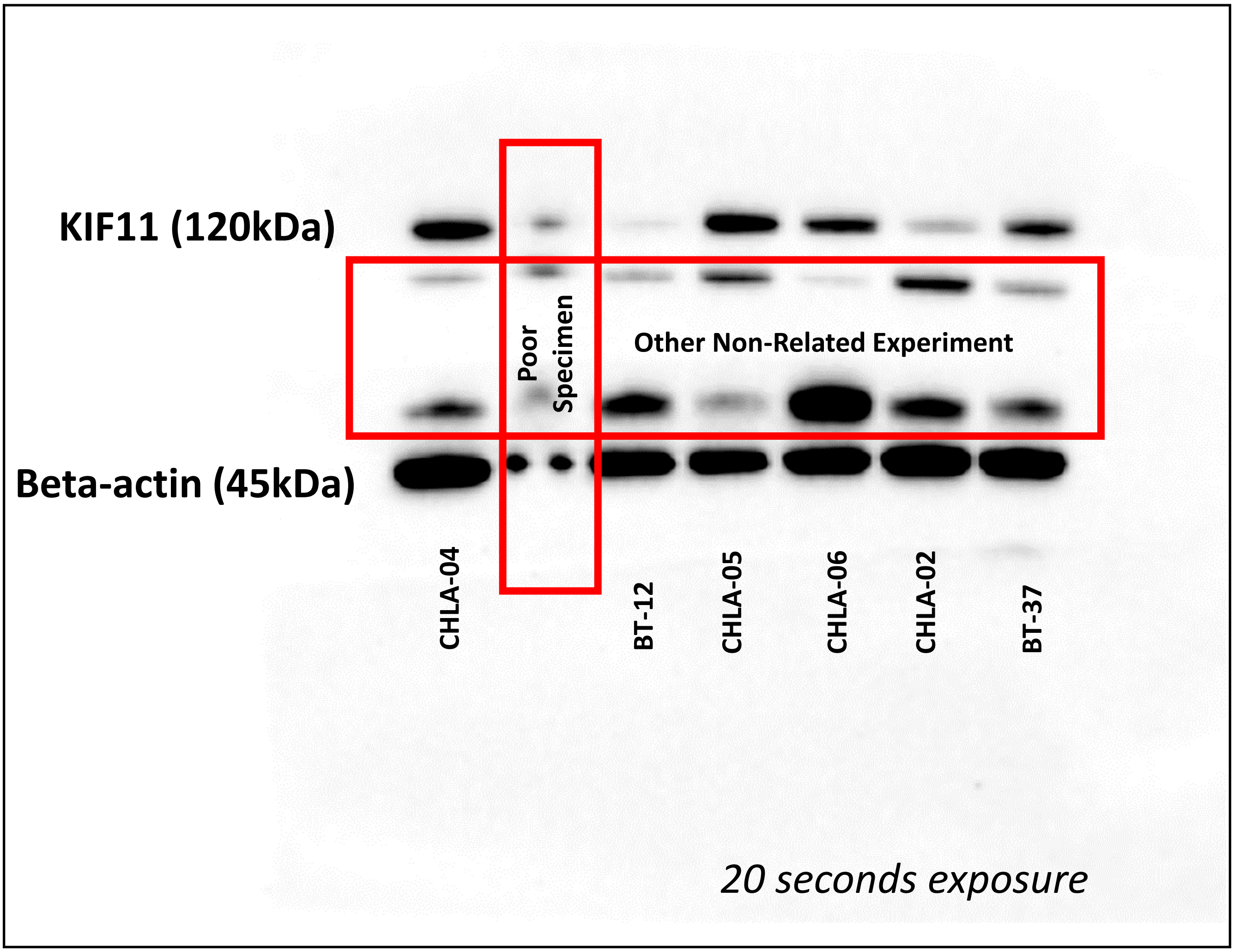

Full Length Blots

Original full length blot for **Fig. S6E.**

CHLA-06 Cells

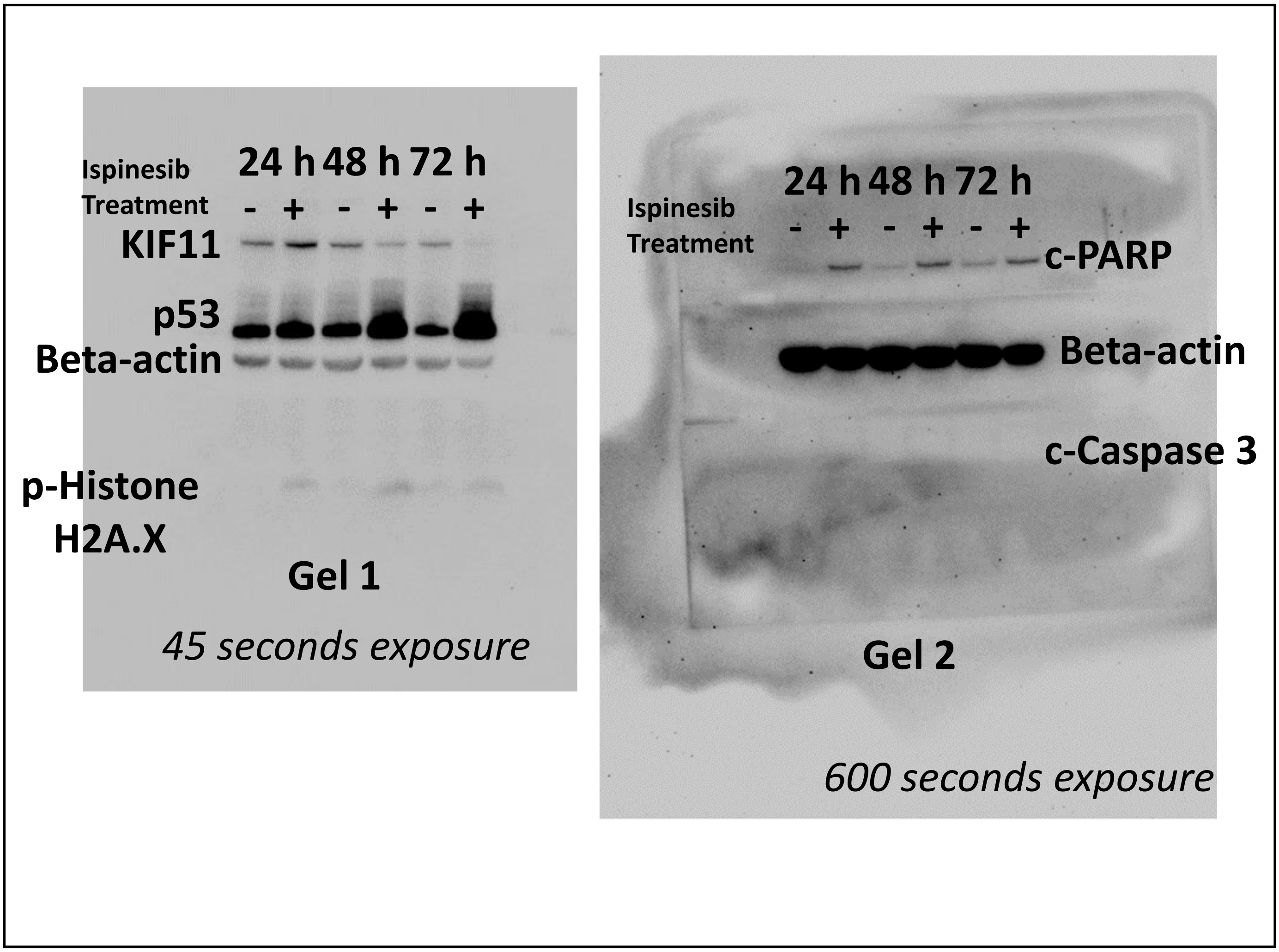

Full Length Blots

Original full length blot for **Fig. S6E.**

CHLA-05 Cells

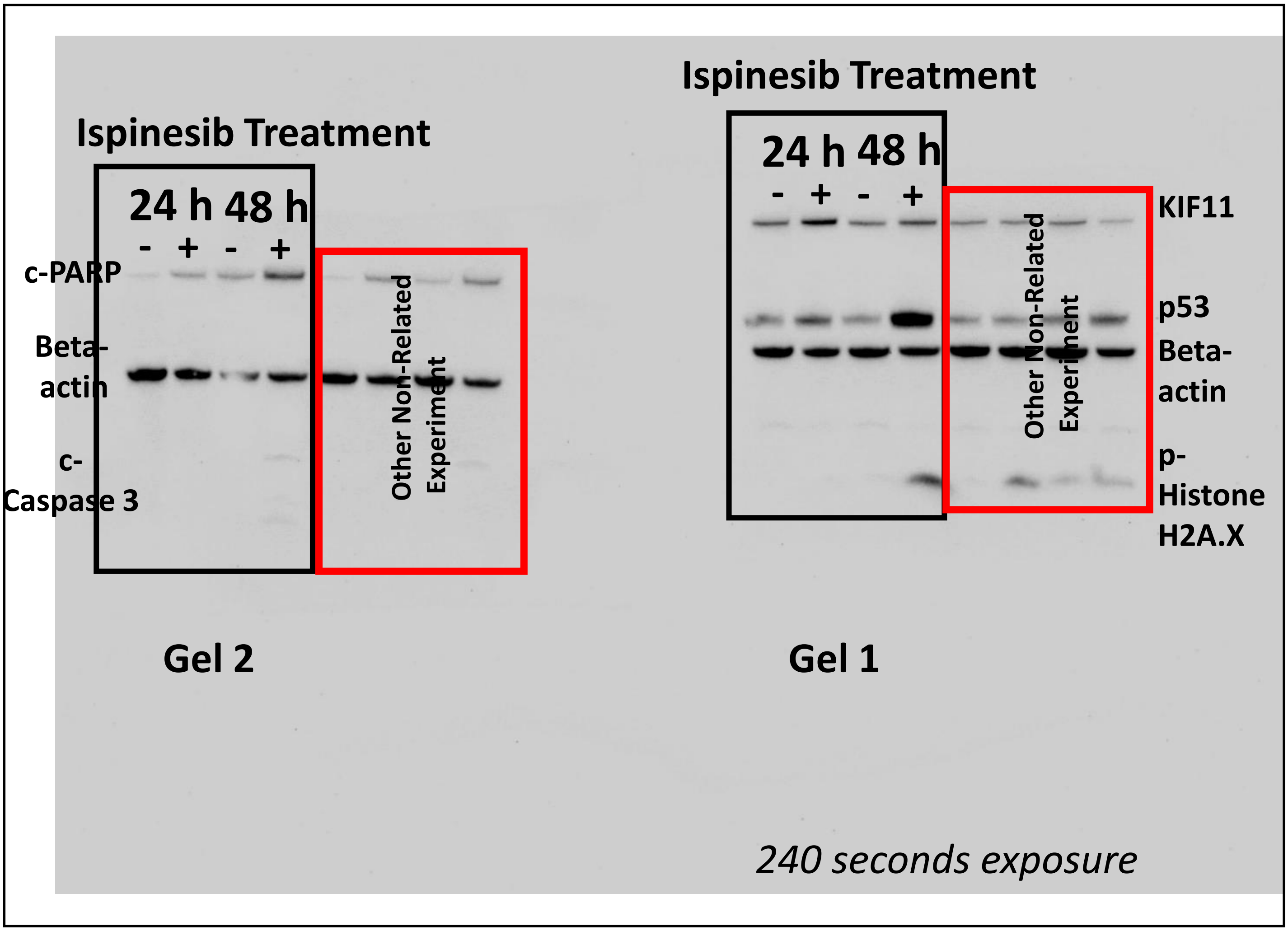

Full Length Blots

Original full length blot for **Fig. S6E.**

BT-37 Cells

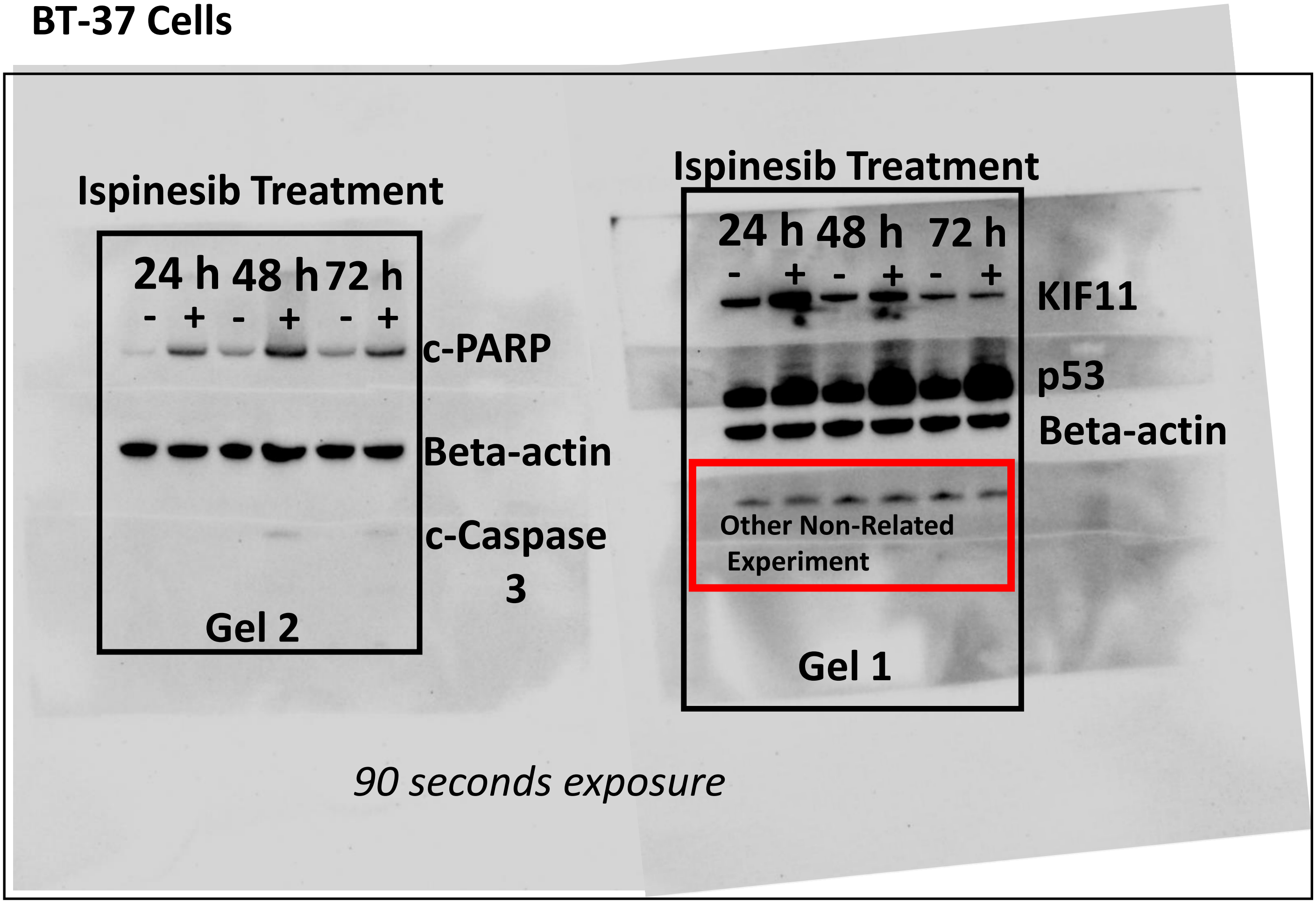

Supplement: Supplementary file 1 — Supplementary Information. [file 41598_2021_91167_MOESM1_ESM.pdf]
